# Social experience and pheromone receptor activity reprogram gene expression in sensory neurons

**DOI:** 10.1093/g3journal/jkad072

**Published:** 2023-03-27

**Authors:** Bryson Deanhardt, Qichen Duan, Chengcheng Du, Charles Soeder, Alec Morlote, Deeya Garg, Aishani Saha, Corbin D Jones, Pelin Cayirlioglu Volkan

**Affiliations:** Department of Biology, Duke University, Durham, NC 27708, USA; Department of Neurobiology, Duke University Medical Center, Durham, NC 27710, USA; Department of Biology, Duke University, Durham, NC 27708, USA; Department of Biology, Duke University, Durham, NC 27708, USA; Department of Biology, University of North Carolina at Chapel Hill, Chapel Hill, NC 27599, USA; Department of Biology, Duke University, Durham, NC 27708, USA; Department of Biology, Duke University, Durham, NC 27708, USA; Department of Biology, Duke University, Durham, NC 27708, USA; Department of Biology, University of North Carolina at Chapel Hill, Chapel Hill, NC 27599, USA; Department of Biology, Duke University, Durham, NC 27708, USA; Department of Neurobiology, Duke University Medical Center, Durham, NC 27710, USA

**Keywords:** Drosophila, olfactory system, transcriptional responses, social experience, pheromone sensing, fruitless

## Abstract

Social experience and pheromone signaling in olfactory neurons affect neuronal responses and male courtship behaviors in Drosophila. We previously showed that social experience and pheromone signaling modulate chromatin around behavioral switch gene *fruitless*, which encodes a transcription factor necessary and sufficient for male sexual behaviors. Fruitless drives social experience-dependent modulation of courtship behaviors and physiological sensory neuron responses to pheromone; however, the molecular mechanisms underlying this modulation of neural responses remain less clear. To identify the molecular mechanisms driving social experience-dependent changes in neuronal responses, we performed RNA-seq from antennal samples of mutants in pheromone receptors and *fruitless*, as well as grouped or isolated wild-type males. Genes affecting neuronal physiology and function, such as neurotransmitter receptors, ion channels, ion and membrane transporters, and odorant binding proteins are differentially regulated by social context and pheromone signaling. While we found that loss of pheromone detection only has small effects on differential promoter and exon usage within fruitless gene, many of the differentially regulated genes have Fruitless-binding sites or are bound by Fruitless in the nervous system. Recent studies showed that social experience and juvenile hormone signaling co-regulate *fruitless* chromatin to modify pheromone responses in olfactory neurons. Interestingly, genes involved in juvenile hormone metabolism are also misregulated in different social contexts and mutant backgrounds. Our results suggest that modulation of neuronal activity and behaviors in response to social experience and pheromone signaling likely arise due to large-scale changes in transcriptional programs for neuronal function downstream of behavioral switch gene function.

## Introduction

Detection of the social environment through pheromone signaling is critical for animals to recalibrate sex-specific behaviors such as mating and aggression (Cushing and Kramer 2005; Curley et al. 2011; Dey et al. 2015; Sethi et al. 2019). It is thought that changes in social environment can modify the regulation of genes necessary for neuronal homeostasis, physiology, and transmission, ultimately affecting circuit function and behaviors (Cushing and Kramer 2005; Flavell and Greenberg 2008; West and Greenberg 2011). Previous studies on the effects of early life experience have identified changes in neuroanatomy, synaptic plasticity, neurotransmission, and gene expression. For example, maternal licking and grooming of pups increase DNA methylation around glucocorticoid receptor gene, leading to long-lasting effects on offspring stress responses and behaviors (Weaver et al. 2004; McGowan et al. 2009; Mifsud et al. 2011). However, transcriptional cascades driving sensory and social experience-dependent modulation of gene expression, circuit function, and behaviors remain unclear.

Identifying gene regulation cascades by which social signals influence neural and behavioral responses requires a model system with well-defined circuits and genetic regulators with roles in neurophysiology, circuit structure, and behavioral function. Circuitry for courtship behavior in *Drosophila melanogaster* is an excellent experimental system to address this question. In Drosophila, male-specific courtship behaviors are governed by a critical transcriptional regulator Fruitless^M^ (Fru^M^), which is encoded by the male-specific alternative splicing of the *fruitless* (*fru*) gene from the P1 promoter (Dickson 2008; Yamamoto and Koganezawa 2013). It is known that Fru^M^ is both necessary and sufficient for male courtship as loss of Fru^M^ in males leads to a loss of male–female courtship (Ryner et al. 1996; Demir and Dickson 2005; Von Philipsborn et al. 2014). Fru^M^ is expressed in approximately 2,000 interconnected neurons throughout the peripheral and central nervous system, and its expression is required for the development, function, and plasticity of the circuit which drives male-specific behaviors (Yamamoto and Kohatsu 2017). In particular, social cues such as pheromones can affect courtship behaviors in males (Kurtovic et al. 2007; van Naters and Carlson 2007; Yamamoto et al. 2013; Dweck et al. 2015; Lin et al. 2016; Yan et al. 2020). Two types of these pheromones, male-specific pheromone *cis*-vaccenyl acetate and non-sex-specific pheromones (such as methyl laurate and palmitoleic acid), activate Fru^M^-positive olfactory receptor neurons (ORNs) expressing Or67d and Or47b receptors, respectively (Kurtovic et al. 2007; Dweck et al. 2015; Lin et al. 2016). These two ORN classes act differently, with Or67d regulating male–male repulsive behaviors and aggression, whereas Or47b driving age and social experience-dependent male copulation advantage (Wang et al. 2011; Dweck et al. 2015; Lin et al. 2016; Sethi et al. 2019).

Previous studies have reported that different social contexts, as well as loss of Or47b or Or67d function, alter the regulation of *fru* transcription, particularly the enrichment of active chromatin marks around *fru* promoters (Hueston et al. 2016; Zhao et al. 2020). In addition, the expression of *fru^M^* isoforms in Or47b and Or67d ORNs affects physiological responses to pheromone ligands and courtship behaviors (Lin et al. 2016; Ng et al. 2019; Sethi et al. 2019; Zhang et al. 2020). It is likely that changes in social context, pheromone signaling, as well as subsequent changes in *fru* regulation, affect the expression of ion channels as well as neurotransmitter receptors regulating neurophysiology. Indeed, Fru^M^ binding is detected upstream of many ion channels and genes controlling neural development and function in the central brain (Neville et al. 2014; Nojima et al. 2014; Vernes 2014). Even though these studies point to the regulation of neuronal and circuit function by Fru^M^, very little is known about how it affects the expression of these target genes, or how pheromone signaling and social experience affect transcriptional programs by modulating Fru^M^.

Here, we performed antennal RNA-seq to determine transcriptional changes in response to social isolation and mutants in pheromone receptors or Fru^M^. Our results showed small modifications to *fru* exon and promoter usage in pheromone receptor mutants. Larger changes were detected in *fru^M^* mutants, suggesting adaptive changes to the *fru* isoform pool in the absence of the male isoforms. We also found that transcriptional programs associated with neural activity and function were altered. Many of the Fru^M^ target genes involved in regulating membrane potentials and synaptic transmission were misregulated in the same direction in *fru^M^* and pheromone receptor mutants. These results uncover a gene regulatory cascade from pheromone receptors to transcriptional programs that alter neuronal responses in different social contexts, potentially through changes in Fruitless function.

## Material and methods

### Fly genetics and genotypes

Flies were raised on standard fly food (containing yeast, cornmeal, agar, and molasses) at 25°C in a 12-hour light/12-hour dark cycle in cylindrical plastic vials (diameter, 24 mm and height, 94 mm). For social isolation (single housing, SH) condition, 80–100 hour-old pupae were separated by sex, and males were placed into individual vials, allowed to eclose alone, and aged to 7 days to deprive flies of pheromone interaction on ORNs. For group housing (GH) condition, 25–30 newly eclosed males were collected and placed into food vials. These were aged to 7 days, and 180 antennae were dissected per sample, for a total of three samples for *w^1118^ GH*, three samples for *w^1118^ SH*, three samples for *Or47b^1^* mutants (*Or47b^1^/Or47b^1^*; *Or47b-GAL4*, *UAS-mCD8GFP/Or47b-GAL4*, *UAS-mCD8GFP*), three samples for *Or67d^GAL4^* mutants (*UAS-mCD8GFP/UAS-mCD8GFP*; *Or67d^GAL4^/Or67d^GAL4^*), and two samples for *fru^LexA^/fru^4–40^* mutants (*w^+^*; *+/+*; *fru^LexA^/fru^4–40^*).

The genotypes used for in vivo imaging validation are listed in Table 1.

**Table 1.** Genotypes used in in vivo validation of expression.

Figure	Type	Genotype	Source
Fig. 6g,g’	Control	*+/+*; *5-HT2A[2048-GAL4]/40XUAS-mCD8GFP*	*5-HT2A[2048-GAL4]*: BDSC 66185
	*Or47b^1^*	*Or47b^1^/Or47b^1^*; *5-HT2A[2048-GAL4]/40XUAS-mCD8GFP*	
Fig. 6h,h’	Control	*dmGlut[0546-GAL4]/UAS-mCD8GFP*; *+/+*	*dmGlut[0546-GAL4]*: BDSC 63397; *Or67d^Z3–5499^*: Dean Smith Lab, UT Southwestern
	*Or67d^Z3–5499^*	*dmGlut[0546-GAL4]/UAS-mCD8GFP*; *Or67d^Z3–5499^/Or67d^Z3–5499^*	
	*fru^LexA^/fru^4–40^*	*dmGlut[0546-GAL4]/UAS-mCD8GFP*; *fru^LexA^/fru^4–40^*	
Fig. 8d,d’	Control	*Jheh3[0892-GAL4]/UAS-mCD8GFP*; *+/+*	*Jheh3[0892-GAL4]*: BDSC 63877; *Or67d^Z3–5499^*: Dean Smith Lab, UT Southwestern
	*Or67d^Z3–5499^*	*Jheh3[0892-GAL4]/UAS-mCD8GFP*; *Or67d^Z3–5499^/Or67d^Z3–5499^*	
	*fru^LexA^/fru^4–40^*	*Jheh3[0892-GAL4]/UAS-mCD8GFP*; *fru^LexA^/fru^4–40^*	
	*Or47b^1^/+*	*Or47b^1^ Jheh3[0892-GAL4]/+*; *40XUAS-mCD8GFP/+*	
	*Or47b^1^*	*Or47b^1^ Jheh3[0892-GAL4]/Or47b^1^*; *40XUAS-mCD8GFP/+*	

### RNA-seq

RNA-seq was performed as described before (Li et al. 2016). Male flies are aged for 7 days and dissected for the third antennal segment (∼180 antennae per genotype). RNA was extracted from dissected tissues samples using Qiagen RNA-easy extraction kit, quantified using a Qubit RNA assay kit, and checked for quality using a High Sensitivity RNA ScreenTape on a TapesStation (Agilent). RNA integrity scores are typically 7.0 and greater. 1 μg of RNA was used to construct libraries for sequencing using a KAPA mRNA library prep kit with polyA RNA selection. Barcoded libraries are sequenced on a Novaseq 6000 SP 50 bp following manufacturer's instructions (Illumina). After demultiplexing, sequence quality was assessed using FASTQC (Version 0.11.9). While there are issues with under-clustering of the samples and unbalanced pools, the data quality was typical for RNA extracted from fresh frozen material. The unbalanced pools resulted in differences in sequencing depth of each sample. The raw data from antennal RNA-seq experiments in this study are already public in GEO (# GSE179213).

### Analysis of RNA-seq data

Once sequenced, the reads are preprocessed with FASTP to remove adaptors and trim/filter for quality. These are mapped to the dm6 reference genome using MapSplice2, with individual mapping rates exceeding 98% in all cases. This raw alignment was deduplicated and filtered for mapping quality and correct pairing; additional alignments are generated to confirm results are robust to mapping ambiguity. Mapped reads are assigned to genes in the annotation using the feature Counts command from the SubRead package (Liao et al. 2014). Differential expression was modeled with DESeq2 using the “apeglm” shrinkage estimator, and data was processed and visualized in R using the tidyverse framework, supplemented with the biomaRt, ComplexHeatmap and UpSet packages. The bioinformatics pipeline was implemented in Snakemake (Köster and Rahmann 2012). Code for the analysis is deposited on GitHub (https://github.com/csoeder/VolkanLab_BehaviorGenetics/tree/master/scripts).

DEXSeq was used to test for differential exon use under models corresponding to those used in differential gene expression (Anders et al. 2012). From the genome-wide test, the *fruitless* locus was examined in particular.

### Statistical analysis

Adjusted *P*-value were directly calculated from DESeq2 or DEXSeq (Supplementary Table 1). Other statistical analysis is described in the legend of corresponding figures.

Specifically, to compare the exon usage in Fig. 4, we also calculated *P*-value from post hoc *t*-tests from raw read counts of independent comparisons of group-housed male antennae to each experimental condition at an individual exon segment (regions 1–22, see Table 2). Even though many exons level differences were significant using this method, adjusted *P*-value from DEXSeq gave rise to fewer significantly altered exon levels.

**Table 2.** *t*-Test *P*-value of fru exons from DEXSeq.

*t*-Test *P*-value	*w^1118^ GH* vs			
Region	*w^1118^ SH*	*Or47b^1^*	*Or67d^GAL4^*	*fru^LexA^/fru^4–40^*
P1 (1)	0.5696	0.0316	0.0619	0.0764
Male (2)	0.6843	0.0292	0.0125	0.0013
Female (3)	0.0697	0.3486	0.6932	0.5993
P2 (4)	Expression too low to calculate			
P6 (5)	Expression too low to calculate			
P3 (6)	Expression too low to calculate			
Exon 7 (7)	Expression too low to calculate			
Exon 8 (8)	Expression too low to calculate			
PD (9)	Expression too low to calculate			
P4 (10)	0.8788	0.0387	0.3002	0.1493
P5 (11)	Expression too low to calculate			
C1 (12)	0.5489	0.0117	0.1050	0.0498
C2 (13)	0.6455	0.0826	0.0007	0.0079
C3 (14)	0.3622	0.1247	0.0399	0.0650
C4 (15)	0.8295	0.3811	0.0704	0.1205
D (16)	0.7090	0.2796	0.2787	0.7042
C5 (17)	0.7575	0.7338	0.2378	0.3174
3′UTR (18)	0.7235	0.0086	0.0754	0.1328
FruA (19)	0.5241	0.0498	0.0171	0.0878
FruB (20)	0.8142	0.4809	0.7873	0.2550
FruMC male (21)	0.8519	0.1819	0.1969	0.1943
FruFC female (22)	0.4168	0.1123	0.8214	0.0223

### Quantitative reverse transcription PCR (qRT-PCR)

The qRT-PCR protocol was modified based on the previous protocol of the Volkan lab (Li et al. 2016). For each genotype (same as RNA-seq), four biological replicates were prepared separately, with each replicate containing 100 antennae from 50 males (7-day old). Antennae were dissected on Flypad and transferred into TRIzol (Invitrogen, 15596026) immediately. Total antennae RNA was extracted using the RNeasy Mini Kit (QIAGEN, 74104) and treated with Dnase I (TURBO DNA-free Kit, Invitrogen, Thermo Fisher Scientific AM1907) to remove genome DNA. cDNA was generated from the reverse transcription of 80–150 ng total RNA using the SuperScript IV First-Strand Synthesis Kit (Invitrogen, 18091050) and poly d(T) as transcription primers. qPCR was performed using the FastStart Essential DNA Green Master kit (Roche, 06924204001) on LightCycler 96 instrument (Roche, 05815916001). Primers used are listed in Table 3. The expression level was calculated by ΔCt method using the *fl(2)d* as the standard gene. The calculation was performed in GraphPad Prism software. One-way ANOVA was used for significance test, followed by multiple comparisons (compare other groups to group-housed wild types *w^1118^ GH*). *P < 0.05, **P < 0.01, ***P < 0.001, ****P < 0.0001.

**Table 3.** Primers sets used in qRT-PCR assays.

Primer names (F: forward; R: reverse)	Sequences
fl(2)d set15 F (exon spanning)	AGAAATCGCAGTCGGAGTT
fl(2)d set15 R	CCTTCTCAAGCGTTTGTATGC
fruM F (exon spanning)	CCCGCATCCCCTAGGTACAA
fruM R	GACTGTTTCGCCCTCGCAGG
dsxM F	GAGCTGATGCCACTCATGTAT
dsxM R	CTGGGCTACAGTGCGATTTA
wkd set34 F	AATGTGCTAAAGGCCTACTC
wkd set34 R (exon spanning)	TGCAGGTATACATCGCACA
ppk25 set11 F (exon spanning)	CTGCAGTATTACAGTCCCTACC
ppk25 set11 R	TCCGGATACTGTGCAGATTG
5-HT2A set15 F (exon spanning)	CCGTTCTTGGTCTGGTCAAT
5-HT2A set15 R	CGTCAATGCGTATGTGGTAAC
dmGlut set45 F	TCCTGAATGCCTACACGATG
dmGlut set45 R (exon spanning)	CACCAACACTGGTTCCCT
Jheh3 set10 F (exon spanning)	GACCGAAATTCAGGGCTTG
Jheh3 set10 R	GGTTAGCATGGGTATAAAGTCG
Lush set4 F (exon spanning)	CTTGTCGGGATACGCATAAA
Lush set4 R	TAAGGCCACATGAACTGC
Obp69a set6 F (exon spanning)	CAGGAGCTTCTGTAGATGTG
Obp69a set6 R	CTCCAACAGTGCTTCCAA

### In vivo validation of gene expression

Fly heads were dissected in cold PBT (phosphate buffered saline with Triton X-100) buffer and were fixed in 4% paraformaldehyde (PFA) on nutator at room temperature for 1 hour. Fly heads were washed three times with fresh PBT, and every wash was 10 min at room temperature. Antennae were dissected from heads and were fixed in 4% PFA on nutator at room temperature for 30 min. Antennae were washed three times with fresh PBT, and every wash was 10 min at room temperature. Antennae were mounted using Fluoromount-G Slide Mounting Medium (SouthernBiotech). Images were taken by Olympus FluoView FV1000 confocal microscope. Controls and experimental groups were imaged by same parameters. Native fluorescence was measured by ImageJ. The fluorescence intensity was defined as the fluorescence of region of interest subtracted by that of the background. Statistical tests were performed in GraphPad Prism software. One-way ANOVA was used for significance test, followed by multiple comparisons (compare other groups to group-housed control). *P < 0.05, **P < 0.01, ***P < 0.001, ****P < 0.0001.

## Results

### Neuronal transcriptional programs are modulated with social isolation and lack of pheromone receptors or Fru^M^ function

**Fig. 1.**
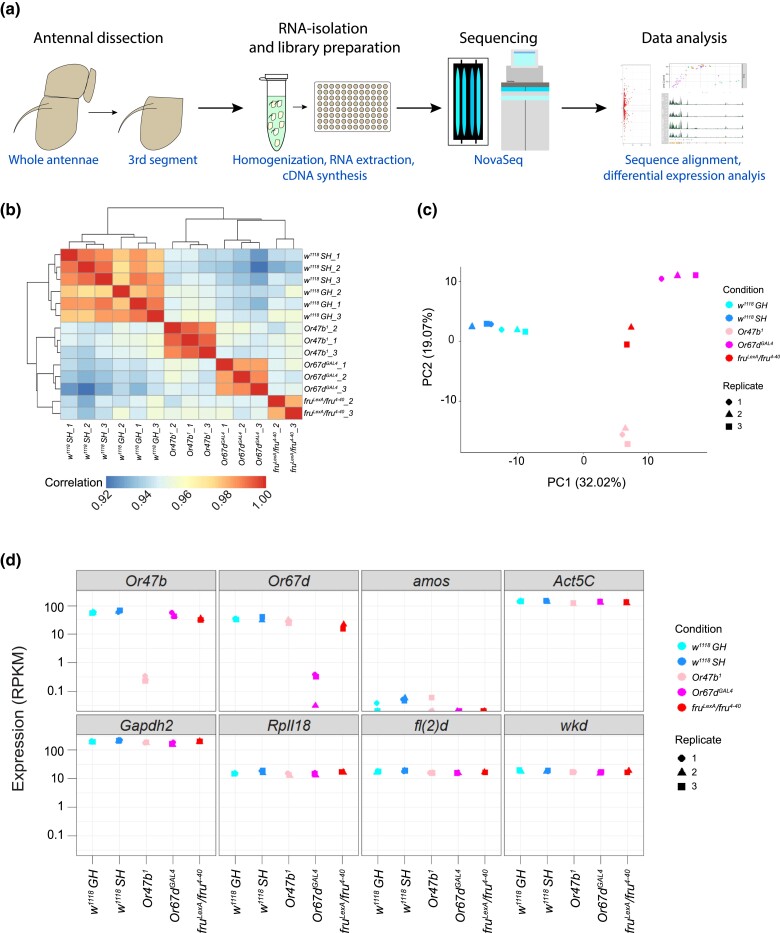
Overview of RNA-seq samples from male antennae. (a) Schematic for antennal RNA-seq workflow. (b–c) Hierarchical clustering based on Pearson's correlation matrix (b) and PCA analysis (c) of transcriptional profiles among biological replicates from antennae of wild-type group-housed (*w^1118^ GH*), single-housed (*w^1118^ SH*), group-housed *Or47b^1^* and *Or67d^GAL4^*, and *fru^LexA^/fru^4–40^* mutant male flies. (d) Transcript levels for several representative negative and positive control genes among all samples.

To identify genes regulated in the peripheral olfactory system by social experience, pheromone signaling, and Fru^M^, we utilized RNA-seq from whole antennae of 7-day old wild-type (*w^1118^*) males that are either group-housed (*w^1118^ GH*) or single-housed (*w^1118^ SH*), as well as group-housed *Or47b* mutant males (*Or47b^1^*), *Or67d* mutant males (*Or67d^GAL4^*), and *fru^M^* mutant males (*fru^LexA^/fru^4–40^*) (Fig. 1a). As noted in the *Material and Methods* and discussed in the *Discussion*, these genetic backgrounds are modestly different compared to typical variation among Drosophila lines (also see Supplementary Fig. 7a and supplemental material *Method* section). Each condition included three biological replicates except for *fru^LexA^/fru^4–40^* with only two (Fig. 1b). Each sample had mapped reads ranging between 24 and 40 million, and hierarchical clustering analysis based on Pearson's correlation between samples showed consistency among replicates within the same genotype (Fig. 1b). Principal component analysis (PCA) also showed the expected grouping of the replicates belonging to the same condition, across the first two principal components accounting for most of the overall variance (32 and 19%) (Fig. 1c). We also found that gene expression changes were more similar among *Or67d*, *Or47b*, and *fru^M^* mutants, compared to grouped or isolated wild-type male antennae (Fig. 1b, c). As expected, expression levels of *Or47b*, *Or67d*, and male-specific *fru* exon were significantly lower in all replicates for *Or47b*, *Or67d*, and *fru^M^* mutants, respectively, though the changes of the whole *fru* gene locus cannot be detected (Fig. 1d and Fig. 4b and Supplementary Fig. 2), validating genotype-specific changes in each condition. In addition, genes known to be absent in adult antennae, such as *amos* (Goulding et al. 2000; Zur Lage et al. 2003; Li et al. 2016), also showed nearly no expression, whereas housekeeping genes, like *Act5C*, *Gapdh2*, *RpII18*, *fl(2)d*, and *wkd*, showed nearly identical expression across all samples (Fig. 1d). These results point to high RNA-seq data quality across sample groups and within biological replicates. The Or47b ORNs were shown to degenerate in 14-day old *Or47b* mutant flies. To test if the transcriptional changes are not due to the decrease of ORN numbers in 7-day old antennal samples, we counted the numbers of Or47b and Or67d ORNs. The total numbers of Or47b or Or67d ORNs were comparable between the control and the *Or* mutants (Supplementary Fig. 1). These results suggest that the changes of gene transcriptional level are mainly due to the loss of Or function, rather than the changes of ORN numbers.

**Fig. 2.**
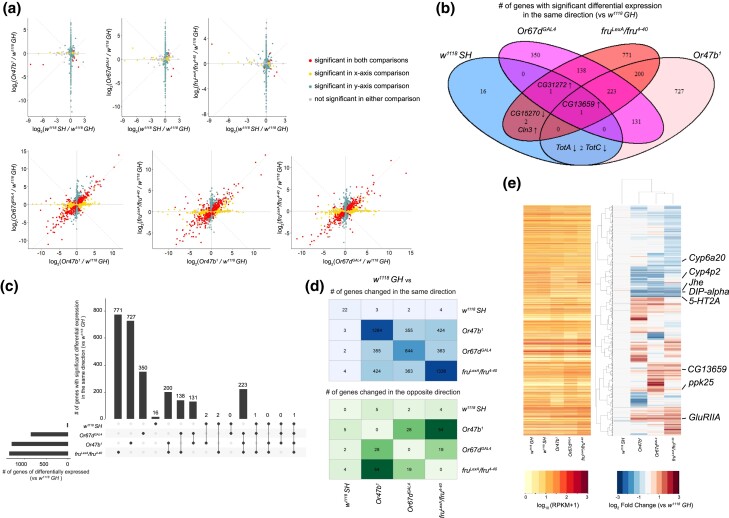
Differentially expressed genes in response to loss of social experience, pheromone receptors, or *fru^M^*. (a) Scatter plot showing the genes that are differentially regulated among social isolation and mutants in pheromone receptors and *fru^M^*. Significance is defined by adjusted *P*-value below 0.01 after applying Bonferroni correction with *n* = 2. (b–c) Venn diagram (b) and UpSet plot (c) comparing differentially expressed genes shared across experimental conditions (only genes changed in the same direction). (d) Numbers of differentially expressed genes with the same (top) direction and the opposite (bottom) direction in pairwise comparison of experimental conditions vs group-housed wild-type samples. In b–d, significance is defined by adjusted *P*-value below 0.01 after applying Bonferroni correction with *n* = 4. (e) Hierarchically clustered heatmaps showing log_2_ fold change compared to group-housed wild-type antennae across all experimental conditions (right) and average mRNA levels (reads per kilobase of transcript, per million mapped reads, RPKM) of replicates within each condition ordered in the same way as log_2_ fold change (left). Only 2,999 genes with at least one significant (adjusted *P*-value below 0.01) change between an experimental condition vs group-housed wild types are shown.

We then ran the differential expression analysis to globally examine the transcriptional changes upon loss of social expression, pheromone sensing, or Fru^M^ function. Compared to group-housed wild-type antennae, social isolation had the least number of significantly altered genes, whereas group-housed *fru^M^* mutants resulted in the highest number (Fig. 2a–c and Supplementary Table 1). Given that *fru^M^* mutants had a smaller sample size in the experiment, this observation needs to be treated with some caution. However, it seems unlikely that less statistical power in the *fru^M^* samples would result in such an excess of differentially transcribed genes. Pairwise comparisons of group-housed wild types to isolated wild types, and *Or47b/Or67d/fru^M^* mutants revealed the genes co-regulated by pheromone receptors and, acknowledging the smaller sample size, *fru^M^* tended to behave in the same direction in the corresponding mutants (Fig. 2a, d), suggesting the shared downstream signaling pathways upon pheromone receptor activation and Fru^M^-dependent regulation. The numbers of genes with significant differential expression in the same direction shared by each condition compared to the group-housed wild types are illustrated in a Venn diagram and Upset plot (Fig. 2b, c and Supplementary Table 2), where genes with overlapping changes in social isolation and *Or47b*, *Or67d*, and *fru^M^* mutants are highlighted. Particularly, only one gene, *CG13659*, an ecdysteroid kinase-like domain encoding gene, is consistently changed across all experimental conditions compared to antennae from the group-housed wild-type males (Fig. 2b).

Hierarchical cluster analysis of differentially expressed genes compared to group-housed wild-type samples showed that the transcriptional changes in *fru^M^* and *Or* mutants were most comparable with one another and most dramatically different from the control (Fig. 2e). Single-housed wild types were most similar to group-housed wild types (Fig. 2e). Cluster analysis identified several genes of behavioral, neurophysiological, and developmental functions such as *Cytochrome p450 6a20* (*Cyp6a20*), *serotonin receptor 2A* (*5-HT2A*), *Juvenile hormone esterase* (*Jhe*), and *Dpr-interacting protein alpha* (*DIP-alpha*) (Fig. 2e) (Liu et al. 2008; Wang et al. 2008; Johnson et al. 2009; Carrillo et al. 2015). Among these, antennal expression of *Cyp6a20*, which is downregulated in *Or47b*, *Or67d*, and *fru^M^* mutants, was previously shown to mediate effects of social experience on male–male aggression (Fig. 2e) (Wang et al. 2008). On the other hand, *Cyp4p2*, which is involved in hormone metabolism and insecticide detoxification (Seong et al. 2018; Seong et al. 2019; Scanlan et al. 2020), is only misregulated in *Or47b* mutants (Fig. 2e). In addition to the downregulated genes, we also found some genes encoding ion channels and neurotransmitter receptors that were significantly upregulated (*ppk25* and *GluRIIA*) (Fig. 2e). The heatmap for gene expression changes revealed gene clusters that were co-regulated by pheromone receptors and Fru^M^, in addition to gene clusters that were uniquely regulated by each OR and Fru^M^; this again highlights that the co-regulated genes tend to change in the same direction in pheromone receptor and *fru^M^* mutants.

### Gene ontology terms for differentially expressed genes in response to lack of social and pheromone signaling highlight neuromodulators

**Fig. 3.**
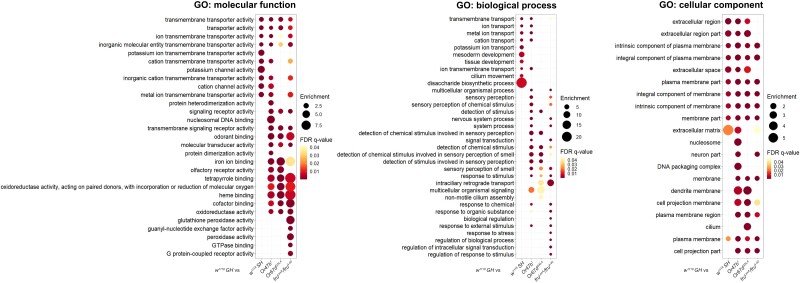
Top enriched gene ontology (GO) terms for differentially expressed genes in response to social experience, pheromone signaling, and Fru^M^ function. The union set of top 10 most significantly enriched GO terms with FDR *q*-value below 0.05 of the differentially expressed genes in each experimental condition is shown. Enriched GO terms were generated by the single ranked gene list with the most significantly changed genes at the top via GOrilla.

Previous work has demonstrated that social experience, pheromone signaling, and Fru^M^ activity can regulate the responsiveness of pheromone sensing ORNs to modify neuronal function and sex specific behaviors (Kurtovic et al. 2007; Wang et al. 2008; Liu et al. 2011; Dweck et al. 2015; Lin et al. 2016; Sethi et al. 2019). To functionally understand system-level changes in gene expression with social isolation, lack of pheromone signaling, and *fru^M^* mutants, we next investigated gene ontology (GO) terms using GOrilla for the list of differentially expressed genes in each experimental condition in pairwise comparisons with group-housed wild types (Eden et al. 2007; Eden et al. 2009) (Fig. 3 and Supplementary Table 3). Many GO terms of molecular function and biological process were commonly affected across multiple experimental groups, suggesting the converging downstream molecular events in response to social experience and pheromone sensing mediated by Fru^M^ activity (Fig. 3). Strikingly, the genes with the altered expression tended to be localized on the cell membrane (Fig. 3, GO: cellular component) and have functions in ion transport across membrane (Fig. 3, GO: molecular function), and appeared to be involved in the process of detecting and responding to olfactory stimuli (Fig. 3, GO: biological process). This supports previous studies in providing a general mechanism for social experience, pheromone receptor signaling, and Fru^M^-dependent regulation of pheromone responsiveness of Or47b ORNs (Sethi et al. 2019; Zhang et al. 2020; Zhao et al. 2020). Furthermore, genes with oxidoreductase activity also had overlapping alterations across *Or47b*, *Or67d*, and *fru^M^* mutants, and many of these appeared to contribute to insect hormone metabolism (Fig. 3, GO: molecular function). Interestingly, previous studies reported that juvenile hormone signaling works together with social experience in olfactory receptor neurons to modulate chromatin around *fru* locus (Sethi et al. 2019; Zhao et al. 2020). Our RNA-seq results also add an additional layer of complexity to hormone-social experience interactions, as social experience and pheromone signaling affect the levels of certain hormones by modifying hormone metabolism dynamics. In summary, social isolation, disrupted pheromone receptor signaling, and lack of Fru^M^ function in peripheral olfactory sensory neurons affect the expression of many genes with roles in diverse aspects of neurophysiology, including neuronal responsiveness, ion transmembrane transport, and beyond.

**Fig. 4.**
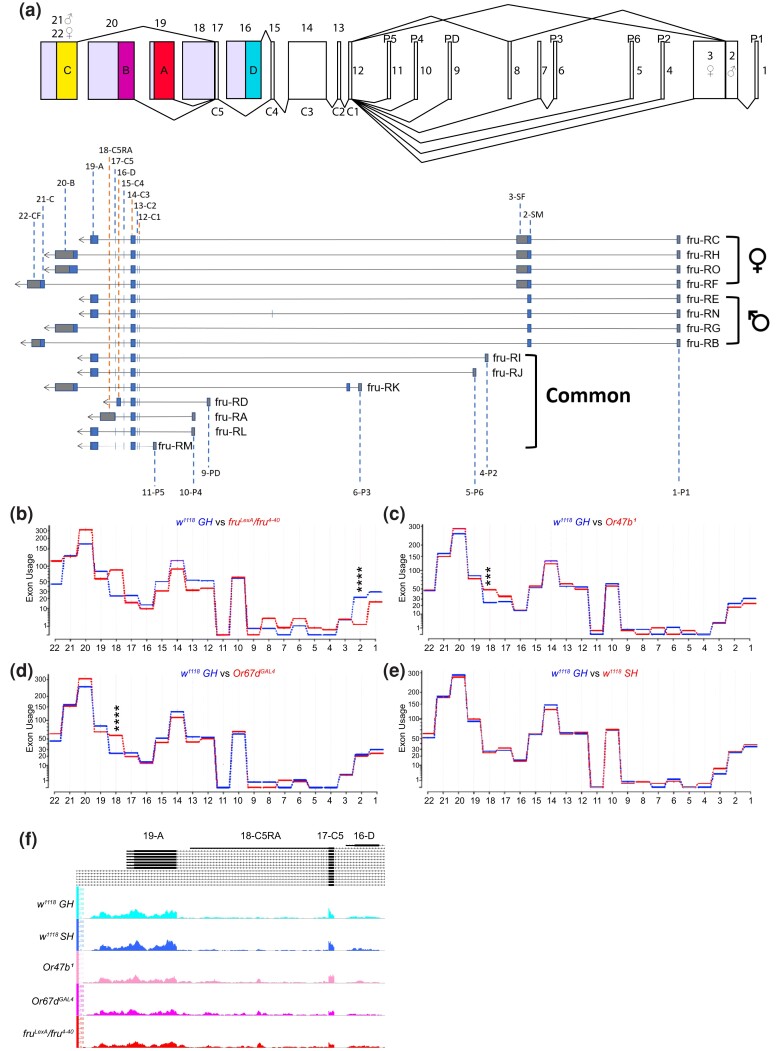
Olfactory stimuli regulate exon usage across the whole *fru* genomic locus. (a) Top: schematic of Fruitless: 1–22 denote each examined region. P1–P6 and PD denote promoters. C1–C5 denote common exons, while a–d denote alternatively spliced 3′ DNA-binding-encoding domains. Bottom: structure of *fru* transcripts with the exon names and numbering used in the schematic above. (b–e) Examination of the usage of various exons in Fruitless to determine distinct changes in *fru^M^* transcripts using DEXSeq, showing exon-by-exon comparison of *w^1118^ GH* vs *fru^LexA^*/*fru^4–40^* (b), *Or47b^1^* (c), *Or67d^GAL4^* (d), and *w^1118^ SH* (e). Adjusted *P*-value was directly performed via DEXSeq (see *methods*—*statistical analysis*). ***P.adjust < 0.001; ****P.adjust < 0.0001. Region 2, *w^1118^ GH* vs *fru^LexA^*/*fru^4–40^*, *P*.adjust = 1.25 × 10^−10^; region 18 (3′UTR), *w^1118^ GH* vs *Or47b^1^*, *P*.adjust = 8.17 × 10^−4^; region 18 (3′UTR), *w^1118^ GH* vs *Or67d^GAL4^*, *P*.adjust = 2.00 × 10^−7^. (f) Read coverage of region 18 (*fru C5RA* exon) in Integrated Genome Browser.

### Loss of pheromone signaling alters *fruitless* splicing patterns and *doublesex* expression

*fruitless* locus (containing multiple promoters, untranslated regions, coding sequences, and denoting regions 1–22 in Fig. 4a) generates multiple alternative splice isoforms for RNAs transcribed from seven promoters (*P1–P6* and *PD*) (Fig. 4a). The transcripts from *fru P1* are alternatively spliced between males and females, where the male isoforms (*fru^M^*) encode functional proteins while female isoforms (*fru^F^*) do not produce any proteins (Dickson 2008; Yamamoto and Koganezawa 2013) (Fig. 4a). The expression of *fru^M^* in males and the absence of functional *fru^F^* transcripts in females help define male and female-specific neuronal pathways as well as the cell-specific expression patterns of genes regulated by Fru^M^. Promoters *fru P2* through *fru P6* produce common isoforms in both males and females that also affect sex-specific activity in courtship circuits of both sexes (Goodwin et al. 2000) (Fig. 4a). *Fru^M^* itself has multiple splicing isoforms that vary in the 3′ end of the mRNA (*fru^MA^*, *fru^MB^*, and *fru^MC^*), which encode Fru^M^ transcription factor proteins with variable zinc finger DNA-binding domains (Goodwin et al. 2000; Neville et al. 2014; Vernes 2014). These regulate different aspects of the circuit controlling courtship behaviors, with Fru^MC^ and Fru^MB^ having the highest overlap behaviorally and Fru^MA^ having little to no effect on courtship (Neville et al. 2014).

We previously showed that social experience and signaling from Or47b and Or67d pheromone receptors alter open chromatin marks around *fru P1* promoter in the male antennae (Zhao et al. 2020). Interestingly, examination of total transcript levels for the entire *fru* gene locus showed little to no difference across experimental conditions (Fig. 1d). These small changes in total transcript levels, despite dramatic changes in open chromatin marks in wild-type SH and mutants in *Or47b*, *Or67d*, and *fru^M^*, prompted us to look at other aspects of gene regulation. It is known that changes in chromatin regulate many aspects of transcription such as transcriptional initiation, elongation, and alternative splicing (Hall and Georgel 2011; Naftelberg et al. 2015). The effects of chromatin on splicing are thought to occur especially because chromatin state alters the speed of RNA Polymerase II (RNAPII), which can lead to splicing mistakes like intron retention or exon skipping (Hall and Georgel 2011).

Given the functional differences in the *fru^M^* isoforms, we predicted that chromatin changes caused by social experience and pheromone receptor signaling could alter *fru* splicing. To explore this, we mapped reads from all experimental conditions to *fru* genomic locus and investigated exon usage levels using DEXSeq (Anders et al. 2012). In general, transcript reads from *fru* locus appear noisier in experimental conditions compared to group-housed wild-type male antennae, with variations in the expression of coding and non-coding sequences (Fig. 4b–e). In *Or47b* mutants, there is a small decrease in *fru P1* promoter (region 1) and male-specific exon (region 2) levels (Fig. 4c, see *methods*—*statistical analysis*). *Or67d* mutants show a small decrease in *fru P1* promoter (region 1) levels and male-specific exon (region 2) (Fig. 4d, see *methods*—*statistical analysis*). The largest change in male-specific exon (region 2) levels is seen in *fru^LexA^/fru^4–40^* allele (Fig. 4b), which has a *LexA* transgene inserted into the first codon of *fru^M^* open reading frame within the male-specific exon (region 2) and a 70-Kb deletion from promoter *P1* to *P3* (Mellert et al. 2010). Surprisingly, *fru^LexA^/fru^4–40^* mutants showed disproportional increase of several 3′-end exons (regions 18, 20, and 22) (Fig. 4b). This suggests the adaptive changes of *fru* isoform pool in the absence of fru male isoforms. *fru P1* promoter (region 1) and the male-specific exon (region 2) are unaltered in socially isolated antennae, yet there is a small increase in the female-specific exon (region 3) (Fig. 4e, see *methods*—*statistical analysis*).

In addition to the first three exons, a non-coding sequence (region 18, *C5RA*) (Fig. 4a), which is only present in the exon *C5* of *fru-RA* transcript (Goodwin et al. 2000), slightly increases in *Or67d* and *Or47b* mutants, as is shown in exon usage quantification (Fig. 4c, d) and read coverage of region 18 locus (Fig. 4f). This transcript encodes a Fru protein that lacks these zinc finger domains but retains BTB/PDZ protein–protein interaction domain (Fig. 4a). It is possible that this isoform can interfere with the transcriptional functions of Fru^M^ proteins by binding and titrating out their interaction partners such as other transcription factors, chromatin modulators, and basal transcriptional machinery (Ito et al. 2012; Chowdhury et al. 2017; Zhang et al. 2018; Sato et al. 2019). Both *fru-RA* and *fru-RL* transcripts use *P4* promoter. Even though we do see small but significant differences in RA-specific 3′UTR in *Or47b* and *Or67d* mutants, the effect on the RL transcript is even smaller compared to the RA transcript. In addition, our qRT-PCR analysis of *fru-RA* exon levels was inconsistent and only sometimes reproduced the RNA-seq results compared to control genes. It is possible due to the difference between the DEXSeq and qRT-PCR analysis, where the DEX-seq measures the overall UTR but the qPCR is only to detect 100–150 bp. The accumulated difference along the whole UTR might not be detectable by qPCR. Given that our RNA-seq is on whole antennal samples, these differences might be larger and more salient at the level of the individual ORNs, and future experiments looking at transcriptional profiles from single-ORN populations or detailed in situ hybridization experiments analyzing expression levels of each *fru* splice isoforms on antennal tissues will help determine the extent and cell-type specificity of these alterations. These results suggest that social and pheromonal cues have modest effects on *fru* exon and promoter usage at the antennal RNA level.

Another sex determination transcription factor known to regulate sex specific behaviors is *doublesex* (*dsx*) (Villella and Hall 1996; Waterbury et al. 1999; Billeter et al. 2006; Kimura et al. 2008; Rideout et al. 2010; Robinett et al. 2010; Dauwalder 2011; Pan et al. 2011; Pan and Baker 2014). *dsx* expression in the antenna is restricted to non-neuronal cells (Robinett et al. 2010). We found that the expression of *dsx* in antenna is significantly increased in *Or* and *fru^M^* mutants, albeit the increase is much more pronounced in *Or67d* and *fru^M^* mutants (Supplementary Fig. 2b). Socially isolation did not alter the expression of *dsx* in antennae (Supplementary Fig. 2b, d). These results suggest that the expression of *dsx* in antennae is repressed by *Or47b*, *Or67d*, and *fru^M^* functions.

Collectively, our results suggest that the expression of two critical transcription factors, Fru and Dsx, which regulate sex-specific behaviors, is modulated by pheromone signaling.

### Bimodal regulation of genes regulating neurophysiology and neurotransmission by Fru^M^ and pheromone receptor signaling

Previous studies have shown that pheromone receptor signaling and social experience-dependent regulation of chromatin and RNAPII enrichment around *fru P1* promoter can ultimately scale and fine-tune behavioral responses to social environment (Sethi et al. 2019; Zhao et al. 2020). Additionally, previous reports on the genome-wide binding profiles for three Fru^M^ isoforms in the central brain revealed isoform-specific differences in target genes that regulate neuronal development and function (Billeter et al. 2006; Neville et al. 2014). Fru^M^ motifs are enriched among regulatory elements that are open in the female but closed in the male, suggesting Fru^M^ functions as possible repressive transcription factor (Brovkina et al. 2021). Functional differences of Fru^M^ isoforms also influence ORN responses to their pheromone ligands (Zhang et al. 2020). Thus, chromatin-based modulation of *fru* levels and splicing with social experience and pheromone signaling can provide a quick way to modulate neuronal physiology and synaptic communication by modifying gene expression programs. Yet, the effect of social experience and pheromone receptor signaling on gene expression programs or the mode of gene regulation by Fru^M^ (as a transcriptional activator, repressor, or both) remains unclear (Dalton et al. 2013; Neville et al. 2014; Vernes 2014).

**Fig. 5.**
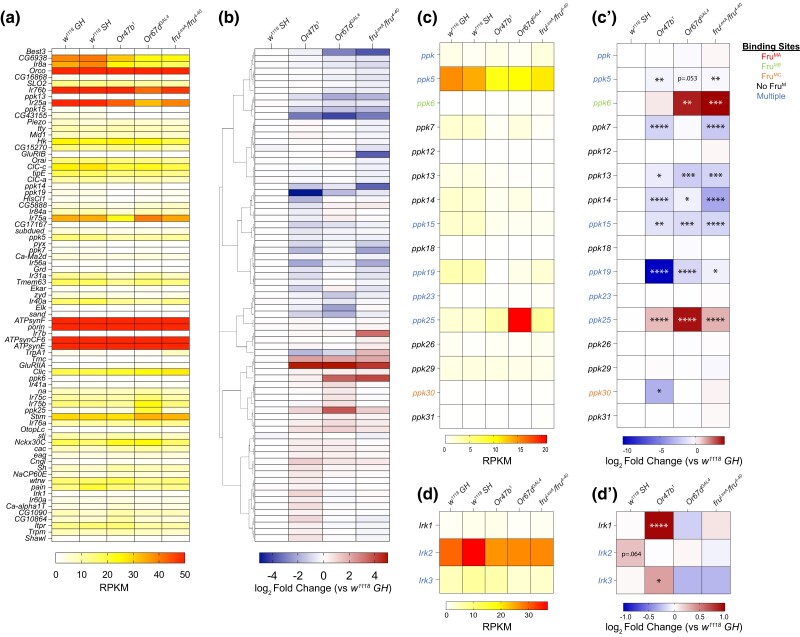
Differentially expressed ion channel-encoding genes in response to social isolation and loss of pheromone receptors or *fru*^M^. (a–b) Examination of GO term: 0005216 (ion channel activity) shows significant changes in various ion channel subclasses. Hierarchically clustered heatmaps showing log_2_ fold change compared to group-housed wild-type antennae across all experimental conditions (b) and average mRNA levels (RPKM) of replicates within each condition ordered in the same way as log_2_ fold change (a). Genes with adjusted *P*-value above 0.01 were filtered out in each experimental condition. (c–c’) RPKM (c) and log_2_ fold change (c’) for *pickpocket* (*ppk*) gene family. (d–d’) RPKM (d) and log_2_ fold change (d’) for *inwardly rectifying potassium channel* (*Irk*) gene family. Adjusted *P*-value was directly performed via DESeq2. *P.adjust < 0.05; **P.adjust < 0.01; ***P.adjust < 0.001; ****P.adjust < 0.0001. Fru^M^-binding information is listed in Supplementary Table 4.

**Fig. 6.**
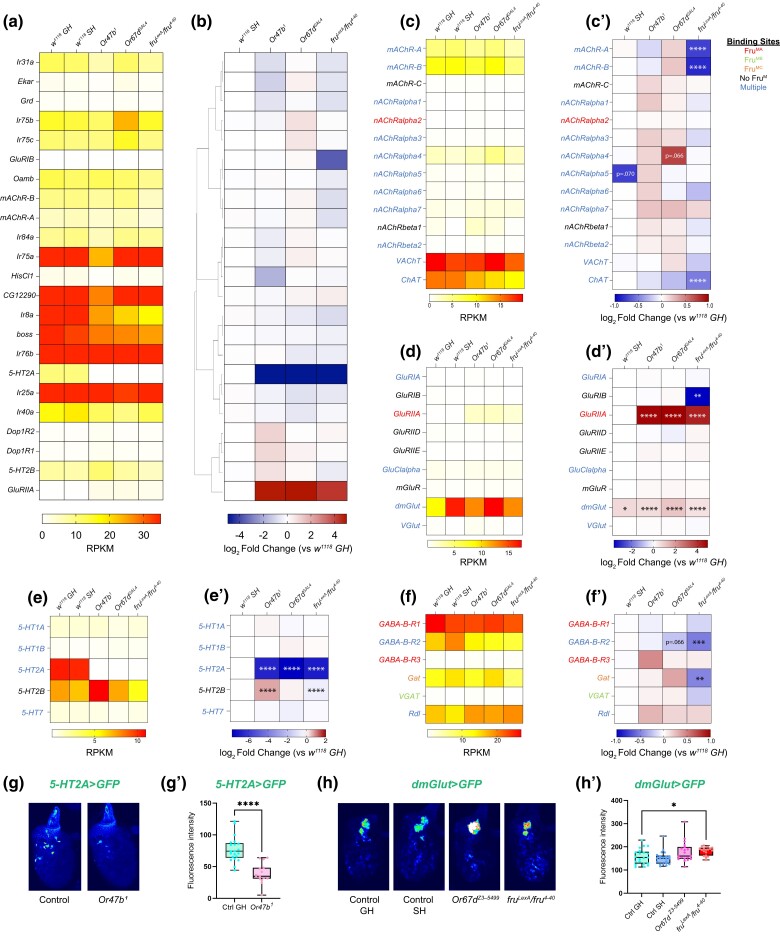
Differentially expressed neurotransmitter receptor and transporter-encoding genes in response to social isolation and loss of pheromone receptors or *fru^M^*. (a–b) Examination of GO term: 0030594 (neurotransmitter receptor activity) shows significant changes in various neurotransmitter activity-associated subclasses. Hierarchically clustered heatmaps showing log_2_ fold change compared to group-housed wild-type antennae across all experimental conditions (b) and average mRNA levels (RPKM) of replicates within each condition ordered in the same way as log_2_ fold change (a). Genes with adjusted *P*-value above 0.01 were filtered out in each experimental condition. (c–c’) RPKM (c) and log_2_ fold change (c’) for acetylcholine-associated genes. (d–d’) RPKM (d) and log_2_ fold change (d’) for glutamate-associated genes. (e–e’) RPKM (e) and log_2_ fold change (e’) for serotonin-associated genes. (f–f’) RPKM (f) and log_2_ fold change (f’) for GABA-associated genes. Adjusted *P*-value was directly performed via DESeq2. *P.adjust < 0.05; **P.adjust < 0.01; ***P.adjust < 0.001; ****P.adjust < 0.0001. Fru^M^-binding information is listed in Supplementary Table 4. (g, h) Confocal images of antenna from WT GH, WT SH, *Or47b^1^*, *Or67d^Z3–5499^*, and *fru^LexA/4–40^* mutants expressing *5HT2A-GAL4* driven *40XUAS-mCD8GFP* (g) and *dmGlut-GAL4* driven *UAS-mCD8GFP* (h). Quantification of fluorescence in images g (g’), and h (h’). (g–g’) *n* = 15–21. (h–h’) *n* = 19–21. (g’, h’) unpaired *t*-test (g’) and one-way ANOVA (h’) were used for significance test, followed by multiple comparisons if necessary (compare other groups to group-housed control). *P < 0.05, **P < 0.01, ***P < 0.001, ****P < 0.0001. Not significant if no * labeled.

As discussed previously, gene ontology analysis of these differentially expressed genes implies that many genes involved in regulating neural activity are regulated by social context, pheromone receptor signaling, and Fru^M^ function. To further investigate this, we specifically focused on genes associated with ion channel activity and/or neurotransmitter regulation (Fig. 5a, b and Fig. 6a, b). We clustered these genes based on their log_2_ fold change in transcript levels compared to group-housed wild types in each experimental condition, while also showing their corresponding expression levels in the antennae (RPKM, reads per kilobase of transcript, per million mapped reads) (Fig. 5a, b and Fig. 6a, b). We also used the single-cell RNA-seq data to provide additional evidence showing the ORN-specific expression patterns of the genes that show differential expression in different social and mutant conditions. We found that many ion channels and/or neurotransmitter receptor-encoding genes showed up/downregulation in response to social isolation and loss of Or47b, Or67d, or Fru^M^ function (Fig. 5a, b and Fig. 6a, b). Within ion channels, two subclasses stood out. These are the Degenerin/Epithelial Sodium Channel (DEG/ENaC) proteins known as pickpockets (Ppks) and inward-rectifying potassium channels Irks. Additional genes also include those encoding calcium channels, for example, *Piezo*, *TrpA1*, and *cacophony* (*cac*) (Fig. 5a, b).

#### Ppk family

We specifically focused on two ion channel families, *pickpocket* family of sodium channels and potassium channels. Recent reports pointed to the function of DEG/ENaC channels known as *pickpocket* family of sodium channels that act in Or47b and Or67d ORNs to regulate responses to their ligands (Zhang et al. 2020). Fru^M^-binding motifs have been identified around many of these *ppk* family members, such as *ppk*, *ppk5*, *ppk6*, *ppk15*, *ppk19*, *ppk23*, *ppk25*, and *ppk30* (Dalton et al. 2013; Neville et al. 2014; Vernes 2014). Both *ppk23* and *ppk25* have been identified as necessary for modulating responses of Or47b ORNs through Fru^MB^ and Fru^MC^ activity, respectively, with Fru^MB^ having an antagonistic effect on physiology in Or67d ORNs (Ng et al. 2019; Zhang et al. 2020). In group-housed wild-type antennae, *ppks* show generally low expression based on our transcriptome analysis as well as recent single-ORN RNA-seq data (Mclaughlin et al. 2021), with *ppk5* displaying the highest levels (Fig. 5c). Many *ppk* genes are differentially regulated in *fru^M^* mutants, in agreement with the existing Fru^M^-binding sites at their promoters. For example, *ppk6 and ppk25* are upregulated in *fru^M^* mutants whereas *ppk5*,*7*,*13*,*14*,*15*,*19* are downregulated. The bimodal changes in *ppk* transcripts in *fru^M^* mutants suggest that Fru^M^ can act as both a repressor and an activator of *ppk* gene regulation. *ppk13*,*14*,*15*,*19*,*25* also show correlated changes in *Or47b* and/or *Or67d* mutants. *ppk6* is strikingly upregulated in both *fru^M^* and *Or67d* mutants, whereas *ppk7* is downregulated in both *Or47b* and *fru^M^* mutants (Fig. 5c’). Of note is the significant increase in *ppk25* expression, especially in *Or67d* mutants, which we also confirmed through quantitative RT-PCR (Fig. 5 and Supplementary Fig. 3c–e). *ppk25* is expressed in Or47b and Ir84 ORNs, but not Or67d ORNs, and has been shown to be downstream of Or47b and Ir84a activity altering their neuronal responses (Lin et al. 2005; Starostina et al. 2012; Ng et al. 2019) (Fig. 5c). The shared and mutant specific patterns of *ppk* gene misregulation in *fru*, *Or47b*, and *Or67d* mutants suggest that lack of pheromone receptor function and Fru^M^ activity alters the expression of *ppk* genes in the antennae that contributes to changes in physiological responses.

#### Irk gene family

Irk gene family encodes 3 inwardly rectifying potassium channels (Irk1–3) with binding motifs for Fru^MA^ identified upstream of *Irk2* and binding of both Fru^MA^ and Fru^MC^ found around *Irk3* (Dalton et al. 2013; Neville et al. 2014; Vernes 2014). Three *Irk* genes are expressed in varying levels in the antennae with *Irk1* having the lowest expression and *Irk2* having the highest expression (Fig. 5d). We found that *Irk1* is upregulated in *Or47b* mutants, whereas *Irk2* trends towards upregulation in response to social isolation (Fig. 5d’).

These results suggest that changes in the transcript levels of Fru^M^-regulated sodium and potassium channels with social isolation and in pheromone receptor mutants may contribute to changes in neuronal responses and behaviors.

#### Regulators of neurotransmission

To ask if social experience, pheromone signaling, and Fru^M^ function regulate genes involved in neurotransmission, we next examined the expression of neurotransmitter receptors, transporters, and enzymes for neurotransmitter metabolism. ORNs in the antennae as well as their projection neuron targets around the antennal lobes are mostly cholinergic (Wilson 2013). In the antennal lobe, it has been shown that local interneurons, which include serotonergic, GABAergic, and glutamatergic interneurons, provide cross talk between synaptic partners in the antennal lobe glomeruli (Chou et al. 2010; Wilson 2013). These neurons form connections with both presynaptic ORNS and their postsynaptic partner projection neurons for modulation of neuronal response across glomeruli (Wang et al. 2003; Olsen et al. 2007; Wilson 2013). These connections are required for fine tuning of signaling at synapses as a way of rapid modulation of neuronal function (Wong et al. 2002; Wang et al. 2003; Olsen et al. 2007; Dacks et al. 2009; Johnson et al. 2009; Sudhakaran et al. 2012; Sizemore and Dacks 2016; Mohamed et al. 2019; Zhang et al. 2019; Suzuki et al. 2020). We found a high expression of choline acetyltransferase (*ChAT*) that catalyzes acetylcholine biosynthesis and *VAChT* that packages acetylcholine into synaptic vesicles, coinciding with their reported cholinergic roles in ORNs. Moreover, we also found relatively high expression of several genes encoding receptors of various neurotransmitters, such as choline, serotonin (5-HT), GABA, and glutamate (Fig. 6c–f’). Many of these genes, such as *nAChRalpha4*/*5*, *5-HT2A*, *5-HT7*, *GABA-B-R2*, and *GluRIIA*, have previously been found to regulate courtship behavior in flies through signaling in the antennal lobe (Becnel et al. 2011; Johnson et al. 2011; Clowney et al. 2015; Suzuki et al. 2020). Interestingly, *GABA-B-R2* was shown to be specifically involved in presynaptic gain control of Or47b ORNs (Root et al. 2008). Additionally, single-cell RNA-seq data shows both broadly expressed neurotransmitter genes like *GluRIIB* and 5-*HT2B*, while others are specific to a subset of ORN classes (McLaughlin et al. 2021) (Supplementary Fig. 4). Overall, many of the genes encoding neurotransmitter receptors show expression changes in different experimental conditions (Fig. 6b).

To focus on genes related to specific neurotransmitters, we did not observe any significant changes in response to social isolation, except for a few genes, like *dmGlut*, which is upregulated compared to the group-housed wild types (Fig. 6d, d’). We again found that loss of Fru^M^ function led to bimodal effects on gene expression (Fig. 6c–f’). Indeed, many of these genes have known Fru^M^ binding to their promoters, including receptors *nAChRalpha1*/*3*/*4*/*5*, G*luRIIA*, *GluClalpha*, *5-HT1A*, *5-HT1B*, *5-HT2A*, and *5-HT7*, and transporters/regulators such as *VAChT*, *ChAT*, and *Gat* (Dalton et al. 2013; Neville et al. 2014; Vernes 2014). Some of these genes display correlated changes between pheromone receptor mutants and *fru^M^* mutants, like *GluRIIA*, *dmGlut*, and *5-HT2A*, suggesting that the effects of pheromone signaling on neurotransmission can act via their influences on *fru* regulation (Fig. 6d–e’). The changes in *5-HT2A* were also validated through qRT-PCR (Supplementary Fig. 4c). In the antenna, *5-HT2A*-*GAL4* and *dmGlut-GAL4* expression is observed in a subset of ORNs (Fig. 6g–h’). Interestingly, Or47b and Or67d ORNs do not express *5-HT2A* reporter (Fig. 6g). In agreement with a decrease in *5-HT2A* transcript levels in the RNA-seq and RT-PCR experiments, *5-HT2A* reporter expression was significantly decreased in *Or47b* mutant antennae (Fig. 6g–g’). On the other hand, *dmGlut* expression in the antennae was upregulated in all conditions compared to group-housed male antennae, generally in agreement with the qRT-PCR validation results (Fig. 6d, d’ and Supplementary Fig. 4d). We also used a *dmGlut-GAL4* to visualize the *dmGlut* expression in vivo and detected the signal in a subset of non-neuronal cells in the antennae, though we only observed the statistically significant increase of the *dmGlut* reporter expression in *fru^M^* mutants (Fig. 6h, h’). Evident changes are also observed in some genes not known to be Fru^M^ targets, for example, *GluRIB* which shows downregulation only in *fru^M^* mutants and *5-HT2B* which shows upregulation in *Or47b* and *fru^M^* mutants (Fig. 6d–e’). These may reflect effects of pheromone receptor signaling independent of Fru^M^ function or indirect effects of Fru^M^ activity. To summarize, the systems-level changes in expression of genes involved in neurotransmission and neurophysiology with social experience and pheromone receptor signaling can modulate ORN responses. In addition, these effects on gene expression with social signals can occur either in a Fru^M^-dependent manner or independently of Fru^M^ in response to other gene regulatory pathways activated by pheromone receptor signaling.

#### Odorant binding proteins

**Fig. 7.**
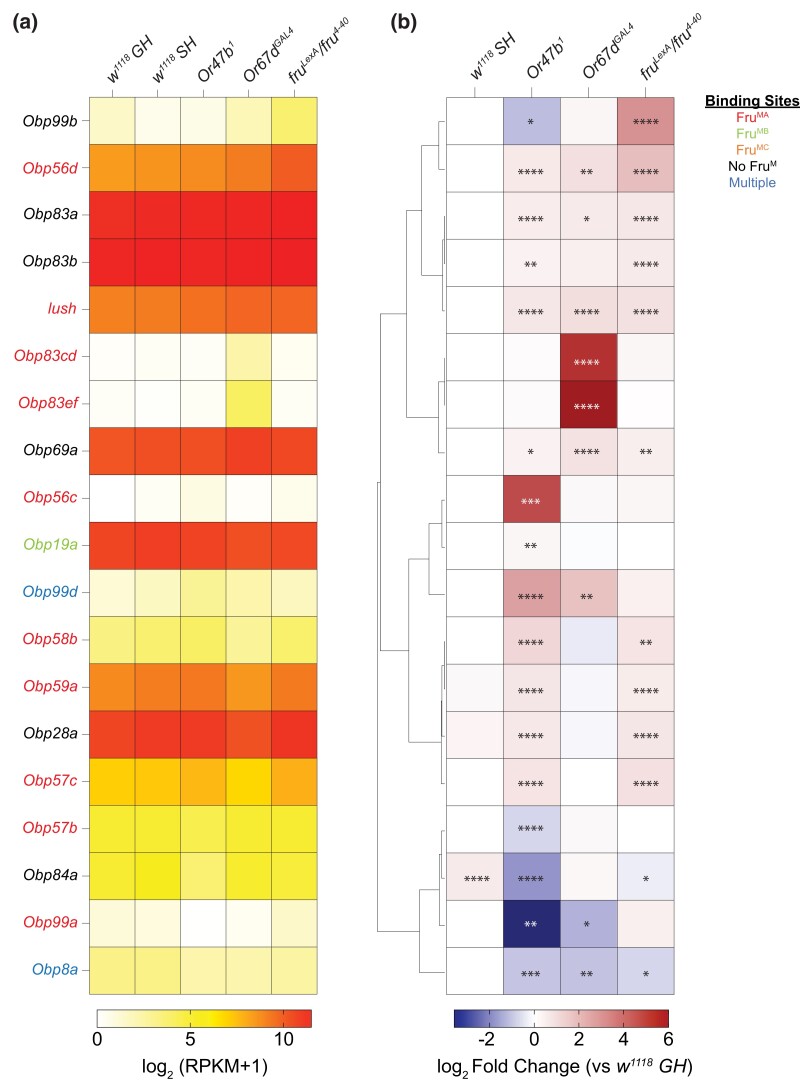
Differentially expressed *odorant binding protein* (*Obp*) genes in response to social isolation, loss of pheromone receptors or *fru^M^*. (a–b) Many *Obp* genes show significant changes. Hierarchically clustered heatmaps showing log_2_ fold change compared to group-housed wild-type antennae across all experimental conditions (b) and average mRNA levels (RPKM) of replicates within each condition ordered in the same way as log_2_ fold change (a). Genes with adjusted *P*-value above 0.01 were filtered out in each experimental condition. Adjusted *P*-value was directly performed via DESeq2. *P.adjust < 0.05; **P.adjust < 0.01; ***P.adjust < 0.001; ****P.adjust < 0.0001. Fru^M^-binding information is listed in Supplementary Table 4.

Analysis of GO terms for molecular function for “odorant binding” highlighted genes encoding odorant binding proteins (Obps) among the significantly altered compared to group-housed wild-type male antennae (Fig. 7). Previous studies using in situ hybridization and transcriptional reporters have shown that Obps are generally produced in the non-neuronal support cells of the antennal sensilla and are secreted into the local hemolymph (Larter et al. 2016). However, our analysis of previously published single-cell RNA-seq data from ORNs revealed that some, but not all, Obps (i.e. *Obp19a*, *Obp28a*, *Obp56a*, *Obp59a*, *Obp69a*, *Obp83a*, *Obp83b*, and *lush*) are abundantly expressed in ORNs in different levels (Supplementary Fig. 5a). Odorants that enter the sensilla through pores are thought to interact with Obps in the hemolymph, which aid with odor binding to receptors on the cilia of ORNs (Larter et al. 2016). Mutants in Obp genes are associated with alterations in the spontaneous or evoked neuronal response dynamics of resident ORNs (Kim et al. 1998; Kim and Smith 2001; Xu et al. 2005; Laughlin et al. 2008; Larter et al. 2016; Scheuermann and Smith 2019).

Analysis of Obp gene expression in the mutant male antennae showed that many Obp transcripts that normally are expressed in trichoid sensilla were increased in the antennae from *Or47b*, *Or67d*, and *fru^M^* mutants (e.g. *Obp83a*, *Obp83b*, *lush*, and *Obp69a*) (McKenna et al. 1994; Pikielny et al. 1994; Xu et al. 2005; Laughlin et al. 2008; Larter et al. 2016; Scheuermann and Smith 2019) (Fig. 7). qRT-PCR from antenna generally corroborates RNA-seq results (Supplementary Fig. 5b, c). Among the Obps that are differentially expressed in mutants, *Obp69a* is particularly interesting as it was previously shown to modulate social responsiveness in Drosophila (Bentzur et al. 2018). In this context, cVA exposure in males as well as activation of Or67d neurons decreases *Obp69a* levels, which in turn alters aggressive behaviors driven by Or67d neurons. In addition, *lush*, *Obp83a*, and *Obp83b* which are also expressed in trichoid sensilla were all shown to regulate odor-evoked response kinetics and spontaneous activity of trichoid ORNs (Kim et al. 1998; Xu et al. 2005; Laughlin et al. 2008; Scheuermann and Smith 2019).

In addition to Obps expressed in the trichoid sensilla, many other Obps also show misregulation particularly in *Or67d* and *Or47b* mutants. For example, in both mutants, *Obp99d* is significantly upregulated; in contrast, *Obp99a* and *Obp8a* show a downregulation (Fig. 7). Even though it is not known which sensilla these Obps are normally expressed in, given the responses, it is likely that they are produced by the non-neuronal cells in trichoid sensilla where Or47b and Or67d ORNs are housed. There are also some Obps that show misregulation only in specific mutants. For example, *Obp83cd*, *Obp83ef*, and *Obp56c* are normally not expressed in the antennae, yet *Obp83cd* and *Obp83ef* show a significant upregulation in *Or67d* mutants, whereas *Obp56c* is upregulated in *Or47b* mutants (Fig. 7). *Obp84a* is the only *Obp* to be upregulated in isolated male antennae and downregulated in *Or47b* mutant antennae (Fig. 7). These results suggest the presence of regulatory interactions between olfactory receptor signaling and neural activity that likely drive activity-dependent homeostasis in Obp levels. Given the role of most Obps in regulating neuronal physiology, it is possible that transcriptional changes in *Obp* genes observed in social isolation as well as pheromone receptor mutants might occur as homeostatic mechanism to compensate for altered neuronal activity and ORN function.

### Pheromone receptor signaling regulates genes involved in hormone metabolism

**Fig. 8.**
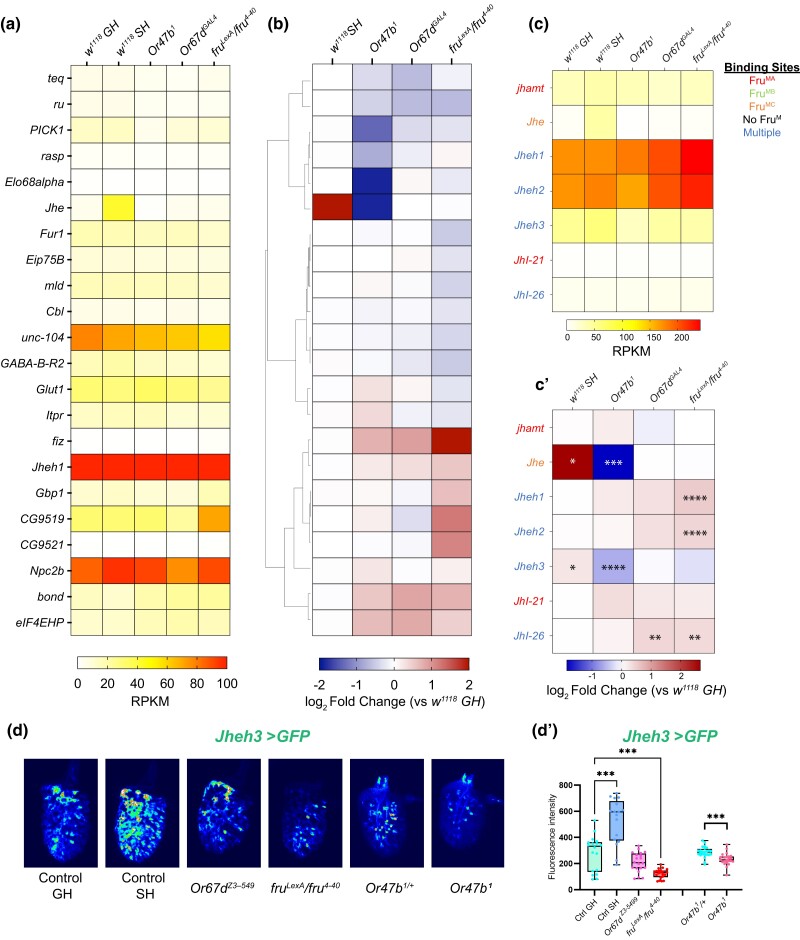
Differentially expressed hormone metabolism genes in response to social isolation and loss of pheromone receptor or *fru^M^*. (a–b) Examination of GO term: 0010817 (regulation of hormone levels) shows significant changes in various hormone metabolism gene subclasses. Hierarchically clustered heatmaps showing log_2_ fold change compared to group-housed wild-type antennae across all experimental conditions (b) and average mRNA levels (RPKM) of replicates within each condition ordered in the same way as log_2_ fold change (a). Genes with adjusted *P*-value above 0.01 were filtered out in each experimental condition. (c–c’) RPKM (c) and log_2_ fold change (c’) for juvenile hormone metabolism-related genes. Adjusted *P*-value was directly performed via DESeq2. *P.adjust < 0.05; **P.adjust < 0.01; ***P.adjust < 0.001; ****P.adjust < 0.0001. Fru^M^-binding information is listed in Supplementary Table 4. (d) Confocal images of antenna from WT GH, WT SH, *Or47b^1^*, *Or67d^Z3–5499^*, and *fru^LexA/4–40^* mutant males expressing *Jheh3-GAL4 UAS-mCD8GFP*. (d’) Quantification of fluorescence in images presented in d. One-way ANOVA was used for significance test, followed by multiple comparisons (compare other groups to group-housed control). *P < 0.05, **P < 0.01, ***P < 0.001, ****P < 0.0001. Not significant if no * labeled. (d–d’) *n* = 17–24.

Hormone signaling is responsible for regulating behavioral and brain states in both vertebrates and invertebrates. For example, in vertebrates, many social behaviors such as aggression, mating, and parenting, are under the control of hormones such as estrogen, testosterone, oxytocin, and vasopressin (Lee et al. 2014; Rilling and Young 2014; Stagkourakis et al. 2020; Liu et al. 2022). In social insects, such as ants, caste-specific behaviors are determined by hormone states, where queen- and worker-like behaviors are associated with ecdysone and juvenile hormone signaling, respectively (Glastad et al. 2020; Gospocic et al. 2021). In Drosophila, juvenile hormone signaling modulates behavioral and motivational states during courtship (Lin et al. 2016; Lee et al. 2017; Zhang et al. 2021). Recent studies have also identified age-related cues such as juvenile hormone (JH) signaling together with social experience to control Or47b neuronal responses to pheromones and courtship behaviors in a Fru^M^-dependent manner (Lin et al. 2016; Sethi et al. 2019; Zhang et al. 2020). JH signaling, concurrent with social experience, modifies chromatin around *fru P1* promoter and ultimately *fru^M^* levels in Or47b ORNs (Zhao et al. 2020). These studies also demonstrated that JH receptor enrichment at *fru P1* promoter increases in socially isolated flies as well as flies with disrupted Or47b signaling (Zhao et al. 2020). As mentioned above, gene ontology analysis of differentially expressed genes in this study also highlights genes involved in hormone metabolism (Fig. 3). Thus, we specifically interrogated the genes regulating hormone levels in pheromone receptor and *fru^M^* mutants (Fig. 8a–c’).

Many of the enzymes involved in juvenile hormone biosynthesis and metabolism, such as juvenile hormone epoxide hydrolases (*Jheh1*,*2*,*3*), *Jhe*, and juvenile hormone acid methyltransferase (*jhamt*), are expressed at varying levels in the antennae (Fig. 8c). These genes are also reported to have Fru^MA^ and Fru^MC^ binding in their upstream regulatory elements (Dalton et al. 2013; Neville et al. 2014; Vernes 2014). Two mostly enriched genes, *Jheh1* and *Jheh2*, show mild upregulation in *fru^M^* mutants but no significant changes in the absence of social cues or pheromone receptor signaling (Fig. 8c, c’). On the other hand, both *Jhe* and *Jheh3* appear to be upregulated in social isolation while downregulated in *Or47b* mutants (Fig. 8c’ and Supplementary Fig. 6b). Throughout the antenna, *Jheh3*-*GAL4* expression is observed in many ORNs (Fig. 8d, d’). In agreement with a transcriptional increase in socially isolated male antennae, we found that *Jheh3* reporter expression was significantly increased in isolated male antennae. On the other hand, in both *Or47b* and *fru^M^* mutant antennae, *Jheh3* reporter expression was significantly decreased in agreement with transcript levels (Fig. 8d, d’). As observed in the RNA-seq, there was no change in *Jheh3* reporter expression in *Or67d* mutants compared to grouped male antennae (Fig. 8d, d’). *Jhe* is of particular interest as Jhe activity is known to be necessary for robust male-specific courtship behaviors and mating success in addition to affecting the abundance of sex-specific pheromones such as 11-cis-vaccenyl acetate in males (Liu et al. 2008; Ellis and Carney 2010). Furthermore, seminal work on Jhe and Jheh3 has shown that these enzymes work together to catabolize JH in *D. melanogaster* (Khlebodarova et al. 1996). These results suggest that social experience and pheromone receptor signaling regulate the expression of JH biosynthetic enzymes. Such changes can modulate juvenile hormone activity by rapidly catabolizing JH in the periphery and affecting downstream target genes, such as *fruitless*.

## Discussion

**Fig. 9.**
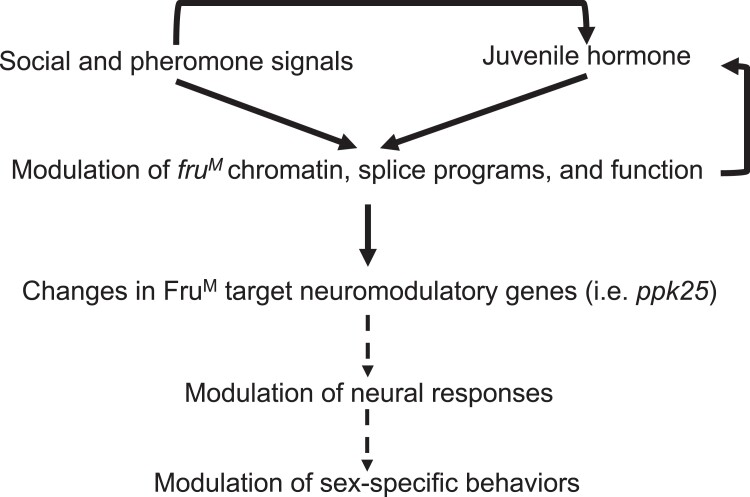
Fruitless-dependent transcriptional cascade that reprograms neural responses and behaviors with social experience, pheromone receptor function, and hormone signaling. Social context and pheromone detection modifies chromatin and transcriptional/splice programs for *fruitless* gene altering its function. This reprograms expression of Fruitless target neuromodulatory genes (i.e. *ppk25*) altering neural physiology and pheromone responses (Ng et al. 2019; Sethi et al. 2019; Zhang et al. 2020). Ultimately, these result in changes in neuronal activity and behavioral modulation (Sethi et al. 2019). It was also shown that juvenile hormone signaling works together with social experience to modulate both ORN physiology and courtship behaviors (Lin et al. 2016; Zhang et al. 2020). At the molecular level, social/pheromonal cues work together with juvenile hormone receptors to modulate transcription fruitless (Zhao et al. 2020). Social context, pheromone receptor, and FruM function also alter the expression of genes involved in juvenile hormone metabolism.

Sensory experience influences many behaviors by modifying neuronal and circuit function (Cushing and Kramer 2005; Curley et al. 2011; Dey et al. 2015; Sethi et al. 2019), yet molecular mechanisms remain largely unknown. Here, we took advantage of the well-characterized system of sex-specific behaviors, governed by the Fru^M^, which acts as a gene regulatory switch for male-specific circuit development, function, and behavior in *D. melanogaster* (Yamamoto 2007; Dickson 2008; Yamamoto and Koganezawa 2013; Yamamoto and Kohatsu 2017). While our sample sizes were modest for *fru* mutants, our results show that social experience and pheromone signaling alter gene expression programs, including modest effects on Fru^M^ splice/promoter usage, ultimately modulating circuit function and behavioral responses (Fig. 9).

As genetic background significantly influences transcriptional profiles, one of the limitations of our transcriptome analysis is that the backgrounds of the mutants and wild type are different. Thus, it is not possible to fully distinguish the effects of background on transcription from effects of mutants used. To further evaluate the scale of potential genetic background influence, we called genetic variants based on the RNA-seq data of this study and calculated the genetic distance among these genotypes together with variation data extracted from the genomes of 18 lines randomly selected from the *Drosophila melanogaster* Genetic Reference Panel (DGRP). As is shown in Supplementary Fig. 7a, while our experimental genotypes are not identical, they are more closely related to each other than those randomly selected DGRP wild-type strains, which are a snapshot of typical genetic variation observed among wild-type isofemale lines. This reflects, in part, a shared genetic ancestry among the laboratory stocks used in this study that resulted from some overlap in the stocks used to make our experimental lines. Secondly, transcript levels for multiple housekeeping genes we analyzed are similar across wild-type and mutant samples (Fig. 1d). We also verified the consistency of these housekeeping genes from additional antennal RNA-seq samples (Supplementary Fig. 7b and Supplementary Table 5). Lastly, we did fully control for the background during *in vivo* confirmation experiments for differentially expressed genes identified by RNA-seq analysis. Using antennal expression patterns of transcriptional reporters for a limited number of relevant genes, we were able to confirm patterns observed in RNA-seq datasets of this study. Collectively, even though the genetic background influences may still exist, the gene sets showing differential expression between *Or/fru* mutants and wild types are largely due to the effects of loss of pheromone receptor or Fruitless function.

Previous studies in Drosophila demonstrated that social experience can modulate Fru^M^-dependent sex-specific behaviors such as courtship and aggression (Curley et al. 2011; Dey et al. 2015; Sethi et al. 2019). For example, social isolation decreases the sensitivity of Or47b neurons to their pheromone ligands in a Fru^M^-dependent manner, which leads to a decrease in male competitive courtship advantage (Sethi et al. 2019). Other studies have also shown that monosexual group housing can decrease aspects of courtship behaviors such as courtship song and circling (Dankert et al. 2009). In addition to courtship, aggression behaviors which are under the control of Or67d and Or65a neurons and Fru^M^ function also change with social experience (Dankert et al. 2009; Liu et al. 2011). For example, social isolation significantly increases male–male aggression (Wang et al. 2008; Dankert et al. 2009). These reports highlight the importance of social experience and pheromone signaling in the execution of sex-specific behaviors.

What are the molecular mechanisms by which Fru^M^ function is altered by social experience? We previously reported that social experience and pheromone receptor signaling alter chromatin states around *fru* P1 promoter (Zhao et al. 2020) to modify *fru* regulation (Hueston et al. 2016; Sethi et al. 2019; Zhao et al. 2020). Surprisingly, as reported in this study as well as in Zhao *et al*. (2020), chromatin alterations at the *fru* P1 promoter in isolated and pheromone receptor mutant male antennae are not accompanied by major changes to transcription, except for a significant decrease in antennal reporter expression driven by *fru P1-GAL4* in *Or47b* mutants (Hueston et al. 2016). Transcriptional regulation of *fru* is complex yielding 15 annotated alternatively spliced isoforms from 7 promoters giving rise to different 3′ sequences which encode variable zinc finger DNA-binding domains of Fru protein (Lee et al. 2000; Meier et al. 2013; Neville et al. 2014; Von Philipsborn et al. 2014). Different Fru proteins regulate unique yet overlapping set of target genes which have binding sites for single or multiple Fru^M^ isoforms (Dalton et al. 2013; Neville et al. 2014; Vernes 2014). Many of these target genes regulate neural development and function. Therefore, changes in *fru* splicing patterns can affect the expression of thousands of genes simultaneously, strongly modulating neuronal responses and circuit outputs in a short period of time. Even though we do detect slight shifts at the level of exon/promoter usage in our transcriptome data, RNA-seq differences from bulk antennal tissues are not dramatic across social conditions and mutants, except for *fru^M^* mutant male antenna. While it is possible that previous chromatin results were noisy, changes in chromatin without associated changes in transcription are a commonly seen phenomenon called “molecular priming” and have been shown in other systems, including in *fru*-positive circuits in the brain (Koike et al. 2012; Jaric et al. 2019; Brovkina et al. 2021). Remarkably, Fru^M^ is expressed in ∼2,000 interconnected neurons highlighting a circuit for courtship behaviors from sensation to action (Yamamoto and Koganezawa 2013; Sato and Yamamoto 2020). This expression pattern allows neural activity-dependent influences on *fru* chromatin and transcription to propagate throughout the whole circuit. In summary, these features make circuit switch gene *fru^M^* an efficient molecular hub onto which many internal and external states act to modulate circuit activity and behavioral outputs by tweaking the levels of transcripts and splice isoforms, leading to a cascade of changes in transcriptional programs.

Each pheromone sensing neuron relays different information about the social environment, which is integrated and processed to output a specific behavior. Likely due to differences in neuronal identity and function, different pheromone receptors have different effects on *fru* chromatin and splice isoforms (Zhao et al. 2020) (Fig. 4). Such sensory stimuli-dependent changes in Fru proteins can alter the expression of downstream genes affecting neuronal activity and function to have rapid, temporary, or lasting effects on neuronal activity and behavioral outputs. These changes are essential for organisms to form short/long-term adaptation to the environment. However, how these different cell types generate these differences in behavioral repertoire via changes in gene expression in the periphery have been largely unknown.

Many of the genes that show differential expression in response to social isolation and disruption of pheromone receptor or Fru^M^ function encode neuromodulators that affect membrane potential, such as ion channels, membrane ion transporters, proteins involved in neurotransmission, and odorant binding proteins (Fig. 3; Fig. 5; and Fig. 6). Among all conditions, social isolation possesses the fewest differentially expressed genes compared to group-housed controls with a small overlap with pheromone receptor and *fru^M^* mutants. This might be due to differences in gene expression changes in response to disruption of evoked activity of pheromone sensing olfactory neurons with socially isolation vs disruption of both spontaneous and evoked activity in pheromone receptor mutants. Loss of Fru^M^ alters the expression of many neuromodulatory genes with known Fru^M^-binding sites in a bimodal way, suggesting that Fru^M^ can act as both an activator and repressor of gene expression. Some of these differentially expressed genes are also altered in pheromone receptor mutants, generally in the same direction (Fig. 2d, e). There are also unique overlaps between *Or47b* and *fru^M^* mutants, between *Or67d* and *fru^M^* mutants, and between *Or47b* and *Or67d* mutants (Fig. 2b, e). Many of these differentially expressed genes are known to harbor binding sites for different Fru^M^ isoforms. These suggest that some of the differentially expressed genes in *Or47b* and *Or67d* mutants are due to Fru^M^-dependent changes, whereas others might be Fru^M^-independent, caused by OR signaling and/or ORN activity.

One functionally relevant gene among the genes that show differential regulation in pheromone receptor and *fru^M^* mutants is the Fru^M^ target gene *ppk25*, which previously was shown to modulate ORN responses in Or47b and Or67d neurons (Ng et al. 2019; Zhang et al. 2020). *ppk25* belongs to a family of sodium channels that serve a variety of functions, from regulation of neural activity to detection of sensory cues. PPK protein complexes generally are composed of multiple subunits encoded by different *ppk* genes. Many *ppk* genes contain binding sites for Fru^M^ isoforms in their promoter regions (Dalton et al. 2013; Neville et al. 2014; Vernes 2014). In addition, a recent study implicated isoform-specific Fru^M^-dependent regulation of *ppk25* and *ppk23* in the modulation of Or47b and Or67d responses (Ng et al. 2019; Zhang et al. 2020). According to the genetic analysis in this study, Fru^MB^ and Fru^MC^ positively regulate the expression of *ppk25* and *ppk23*, respectively. There are apparent discrepancies with this interpretation and transcriptome data from our study, as well as others (Li et al. 2020; McLaughlin et al. 2021). While our transcriptome analysis agrees with a regulatory role for Fru^M^ in *ppk25* gene regulation, the regulatory mode is repressive; that is, *ppk25* expression is upregulated in *Or47b*, *Or67d*, and *fru* mutants. This type of repressive role for Fru^M^ in transcription also is in consensus with previous studies demonstrating Fru^M^ interactions with transcriptionally repressive histone-modifying enzymes such as HDAC1 (Ito et al. 2012; Ito et al. 2013). In addition, we are not able to detect any transcripts for *ppk23* in the antennae, and the expression of *ppk23* does not change in *Or47b*, *Or67d*, and *fru^M^* mutants. Instead, we noticed that other *ppk* genes such as *ppk6*,*7*,*13*,*14*,*15*,*19* are altered in different mutant conditions. Fru^M^ seems to have a bidirectional role in regulating *ppk* gene expression, where it activates the expression of a subset of *ppk* genes (*ppk7*,*13*,*14*,*15*) while repressing the expression of others (*ppk6* and *ppk25*). One way to reconcile these differences is that multiprotein PPK complexes composed of combinations of different PPK subunits and the stoichiometric levels of each *ppk* transcript in a given neuron can determine channel function. For example, misexpression of *ppk23*, which normally is not expressed in the antennal ORNs, can interfere with PPK channel function by disrupting the existing functional complexes in a given neuron, or forming new PPK complexes, thus affecting physiological properties. Another possibility is that the transcriptional changes in *fru^Lex^/fru^/4–40^* mutant are an output for eliminating all *fru^M^* transcripts, thus masking individual effects of each *fru^M^* isoform, such as *fru^MA^*, *fru^MB^*, or *fru^MC^*. And finally, it is also possible that the slight upregulation of *ppk25* in *Or47b* and *fru^M^* mutants as well as large changes in *Or67d* mutants may be due to global *fru^M^* changes in the whole antennae, or through retrograde neuromodulatory signaling from the antennal lobe.

Antennal sensilla contain cell types other than ORNs, such as glia-like cells and support cells of sensillum, as well as epithelial cells. Since our transcription data is from the whole antennae, one possibility we cannot exclude is that differences in antennal gene expression in different genetic and social conditions are readouts from non-neuronal cells or other ORNs. Even though we anticipate the immediate effects of *Or67d* and *Or47b* mutants to happen in the ORNs expressing these two receptors, signals from ORNs can lead to secondary changes in gene expression in non-neuronal cells within the sensillum (Su et al. 2012). This also brings to light a general issue with bulk tissue where large cell-type-specific changes may be masked by cell-nonautonomous changes in gene expression in others cell types, as well as retrograde feedback signaling within olfactory circuits. Regardless, our data shows that many of the differentially expressed genes encode regulators of neuronal function and physiology. This increases the likelihood that the transcriptional changes in response to social and pheromonal cues are happening mostly in the neurons that respond to social cues, such as Or47b and Or67d ORNs. Future single-cell chromatin and transcription profiles from Fru^M^-positive neurons in the antenna and brain will provide deeper insights to neuron-specific changes in gene regulation from the peripheral to the central nervous system that modulate circuit function in response to social cues.

Part of the transcriptional effects can also be exerted downstream of changes in *dsx* levels seen in pheromone receptor and *fru* mutants. Given the upregulation of *dsx* levels in pheromone receptor and *fru* mutants suggests the possibility that some of the social experience- and neural activity-dependent transcriptional changes might also arise from increased Dsx. Dsx expression is restricted to non-neuronal cells in the antenna (Robinett et al. 2010). Similarly, genes affecting neural activity such as *Obps* and some neurotransmitter receptors, which function to alter both spontaneous and evoked activity of ORNs, are also expressed in non-neuronal cells in addition to the ORNs in the antennae (McKenna et al. 1994; Kim et al. 1998; Kim and Smith 2001; Larter et al. 2016). The social experience and pheromone signaling-dependent misregulation of these genes point to adaptive homeostatic mechanism within local sensilla that can contribute to modulation of neuronal activity.

Lastly, in addition to the transcriptional changes occurring in neural activity programs, genes regulating juvenile hormone metabolism are also modified with social context and pheromone receptor and *fruitless* mutants. Social experience works together with juvenile hormone signaling to modulate responses of pheromone sensing neurons in a Fru^M^-dependent manner (Sethi et al. 2019). These contribute to modification of competitive copulation advantage of males in different population densities and different ages as well as regulating overall courtship. At the molecular level, social/pheromonal cues work together with juvenile hormone receptors to modulate chromatin around *fruitless* P1 promoter and its transcription (Zhao et al. 2020). Juvenile hormone acts as a repressor of *fru* expression, and social experience converts it to an activator. In the same study, we showed that social isolation and disruption of Or47b signaling increase the accumulation of juvenile hormone receptor at the *fru* P1 promoter and juvenile hormone response elements. This might be due to changing levels of juvenile hormone since our results show that expression of genes involved in juvenile hormone metabolism are altered in social isolation, and mutants in pheromone receptors and *fru*. The findings in our study together with results from previous studies suggest the presence of interconnected gene regulatory networks among social/pheromone signaling, hormone signaling, and Fru^M^ function in neural and behavioral modulation (Fig. 9).

Social isolation is known to affect a wide range of brain functions and behaviors, such as aggression, attention, depression, and anxiety. Overall, this study highlights the shared transcriptional changes in master behavioral regulators and their target neuromodulatory genes providing a molecular mechanism that alter neural responses with social experience and pheromone sensing.

## Supplementary Material

jkad072_Supplementary_Data

## Data Availability

All relevant data are within the paper and its supporting information files. The raw sequencing data are accessible in GEO (# GSE179213). Code for the analysis is deposited on GitHub (https://github.com/csoeder/VolkanLab_BehaviorGenetics/tree/master/scripts). Supplemental material (Supplementary Figs. 1–7; Suplementary Table 1 contains DESeq2 results and original count/RPKM matrix of all mapped genes in each sample; Supplementary Table 2 contains gene list in Venn diagram of Fig. 2b; Supplementary Table 3 contains detailed GO term analysis results, related to Fig. 3; Supplementary Table 4 contains Fru-binding site information for the genes in Figs. 5–8; Supplementary Table 5 contains RPKM matrix of antennal RNA-seq samples from a different study, related to Supplementary Fig. 7b) available at G3 online.
