# Supplementary material for: Social experience and pheromone receptor activity reprogram gene expression in sensory neurons
Source: G3 (Bethesda). 2023 Mar 27;13(6):jkad072. doi: 10.1093/g3journal/jkad072 (PMC10234412; doi:10.1093/g3journal/jkad072)
Supplement: jkad072_Supplementary_Data [file jkad072_supplementary_data.zip › Supplemental_Material_G3-2022-404026.pdf]

Supplemental Material

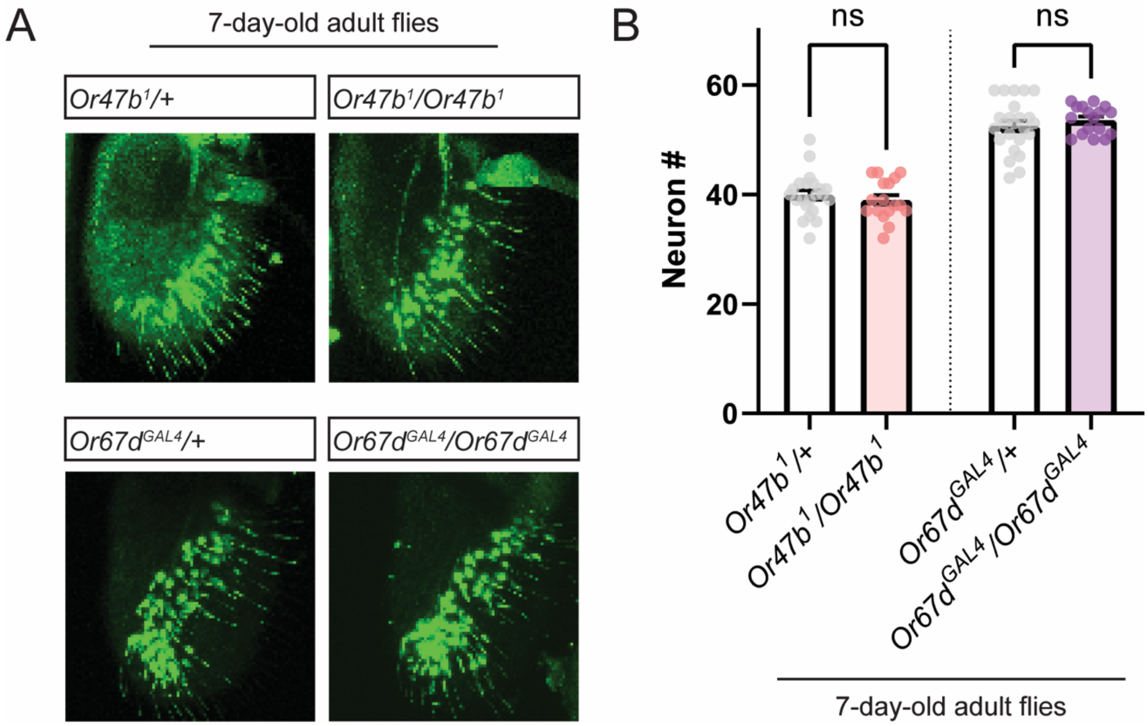

Figure S1. ORN numbers of control and mutants. (A) *Or47b-GAL4* driven *UAS-mCD8GFP* expression in 7-day old male antennae from control and *Or47b* mutants (top), and *Or67d*^{GAL4} driven *UAS-mCD8GFP* expression in 7-day old male antennae from control and *Or67d* mutants. **(B)** Statistics of numbers of Or47b and Or67d ORNs in both control and *Or* mutants. Unpaired t test was used to compare the neuron number between *Or* mutants and control heterozygotes. ns, not significant.

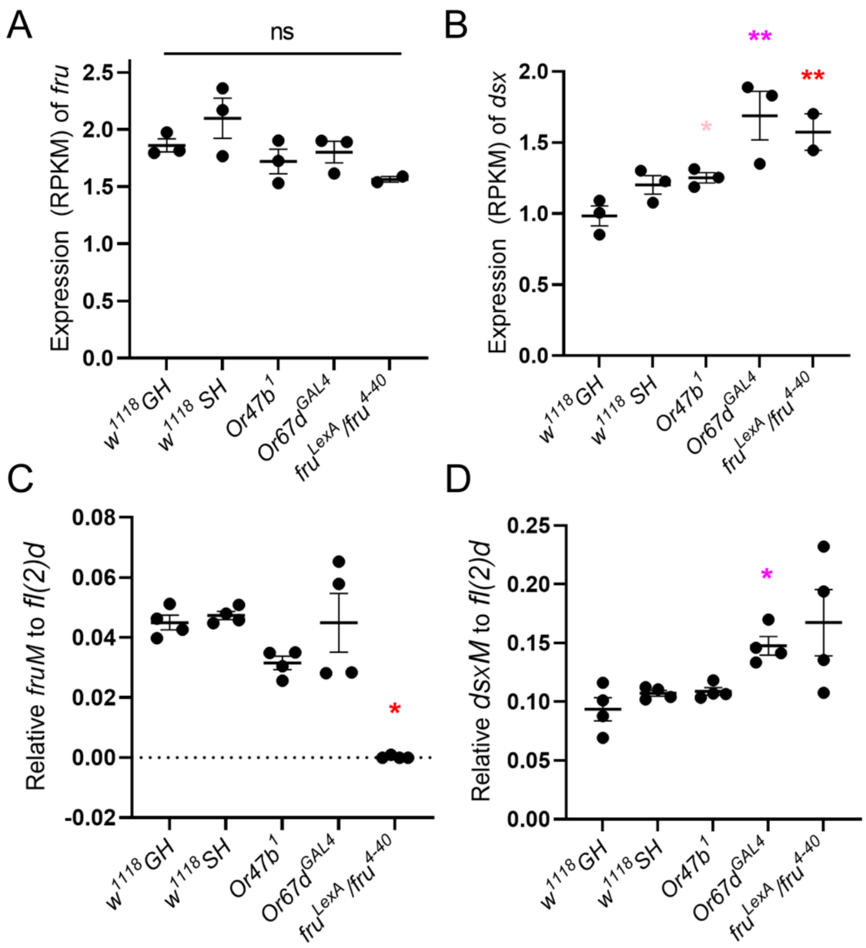

Figure S2. Validation of *fru* and *dsx* male specific exon expression in male antennae of WT and mutants. (A-B) RPKM of *fru* and *dsx.* Adjusted p-value was directly performed via DESeq2. **(C-D)** Quantitative RT-PCR validation of *fru* and *dsx* male specific exon expression. **(C)** The expression of fru^M was not detected in *fru* mutants and did not show significant difference in other *Or* mutants or SH WT. **(D)** The dsx^M expression showed significant increase in *Or67d* mutant male antennae. **(C-D)** One-way ANOVA was used for significance test, followed by multiple comparisons (compare other groups to w^{1118} *GH*). * $p<0.05$, ** $p<0.01$, *** $p<0.001$, **** $p<0.0001$. not significant if no * labeled.

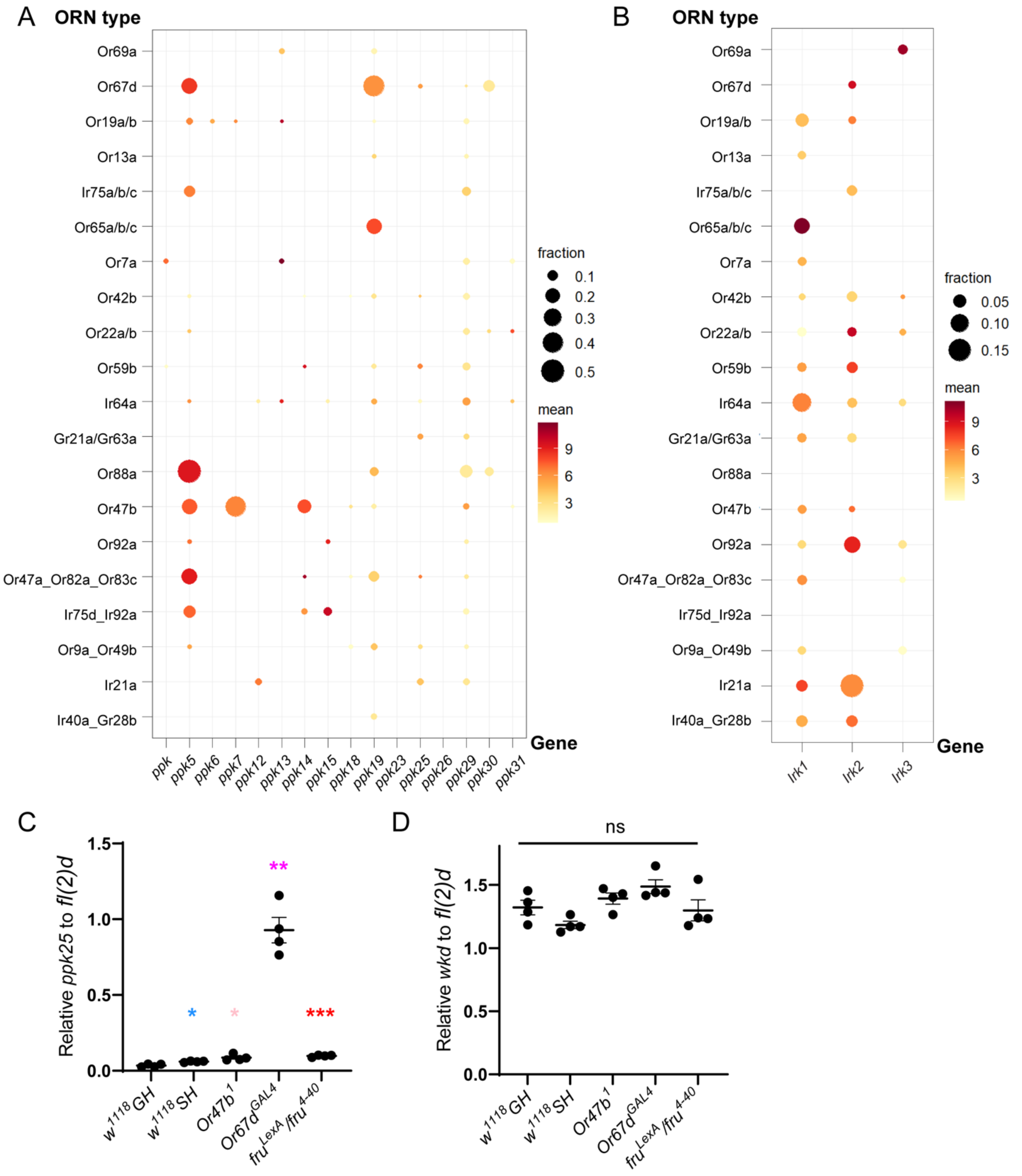

Figure S3. Validation of *ppk* expression across ORN classes and in mutants. (A-B) ORN class-specific expression of *ppk* and *Irk* family genes based on single-cell RNA-seq datasets from the adult ORNs (MCLAUGHLIN *et al.* 2021**)**. Size of each circle

indicates the fraction of positive cells (log_2(CPM+1)>0.5) and color intensity indicates the mean expression (log_2(CPM+1)) of all positive cells. **(C-D)** Quantitative RT-PCR validation of *ppk25* expression **(C)** and a negative control gene *wkd* expression **(D)** from antennae of grouped and socially isolated wild types, *Or47b* mutants, *Or67d* mutants, and fru^M mutants normalized to *fl(2)d*. *fl(2)d* and *wkd* were selected based on their near-identical expression level across all conditions from the RNA-seq results and thus used as loading and negative control genes. One-way ANOVA was used for significance test, followed by multiple comparisons (compare other groups to w^{1118} *GH*). * $p<0.05$, ** $p<0.01$, *** $p<0.001$, **** $p<0.0001$. ns, not significant.

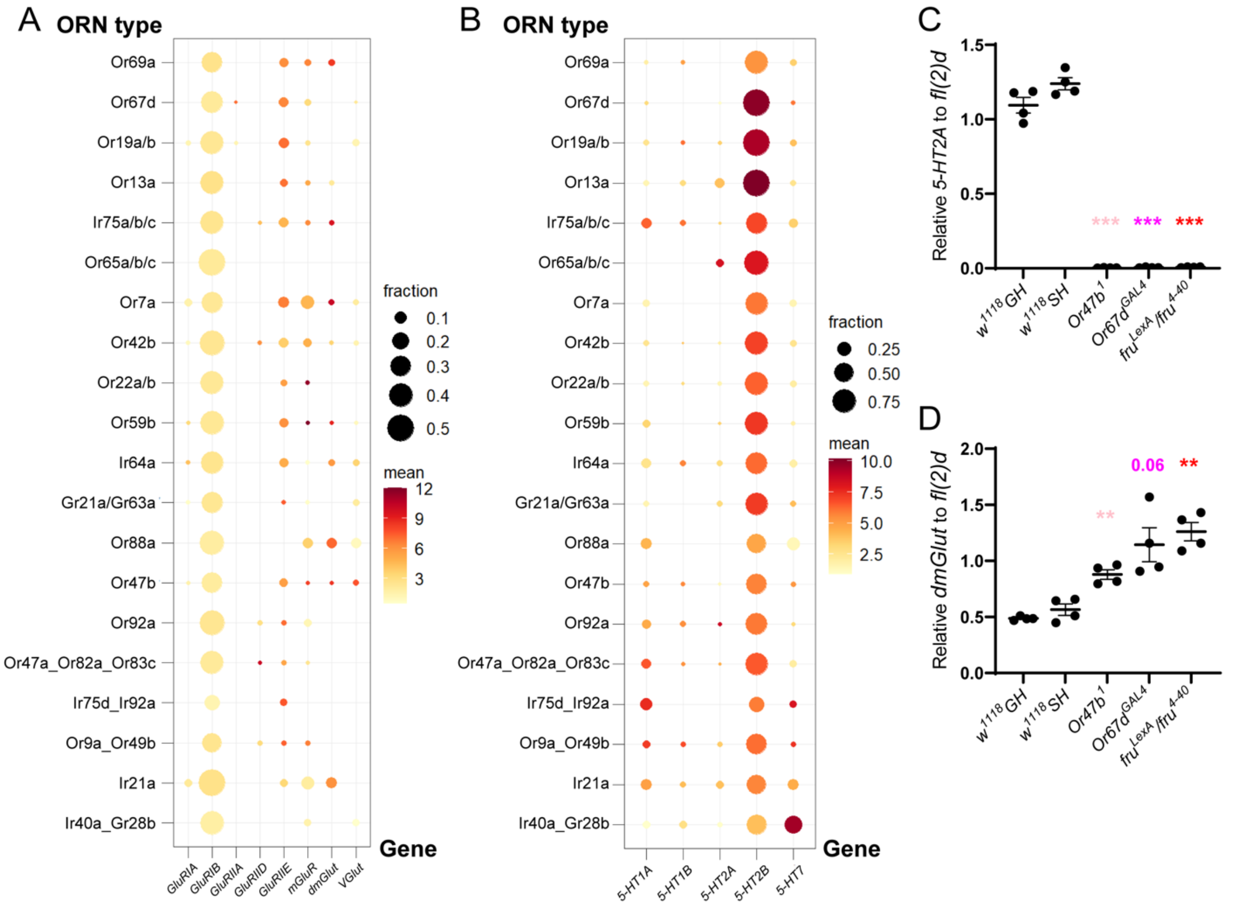

Figure S4. Validation of neurotransmitter receptor expression across ORN classes and in mutants. (A-B) ORN class-specific expression of serotonin and glutamate receptors based on single-cell RNA-seq datasets from the adult ORNs (McLaughlin *et al.* 2021)**.** The size of each circle indicates the fraction of positive cells ($\log_2$(CPM+1)>0.5) and color intensity indicates the mean expression ($\log_2$(CPM+1)) of all positive cells. **(C-D)** Quantitative RT-PCR validation of *5-HT2A* **(C)** and *dmGlut* **(D)** expression from antennae of grouped and socially isolated wild types, *Or47b* mutants, *Or67d* mutants, and fru^M mutants normalized to *fl(2)d*. One-way ANOVA was used for significance test, followed by multiple comparisons (compare other groups to w^{1118} *GH*). * p<0.05, ** p<0.01, *** p<0.001, **** p<0.0001. not significant if no * labeled.

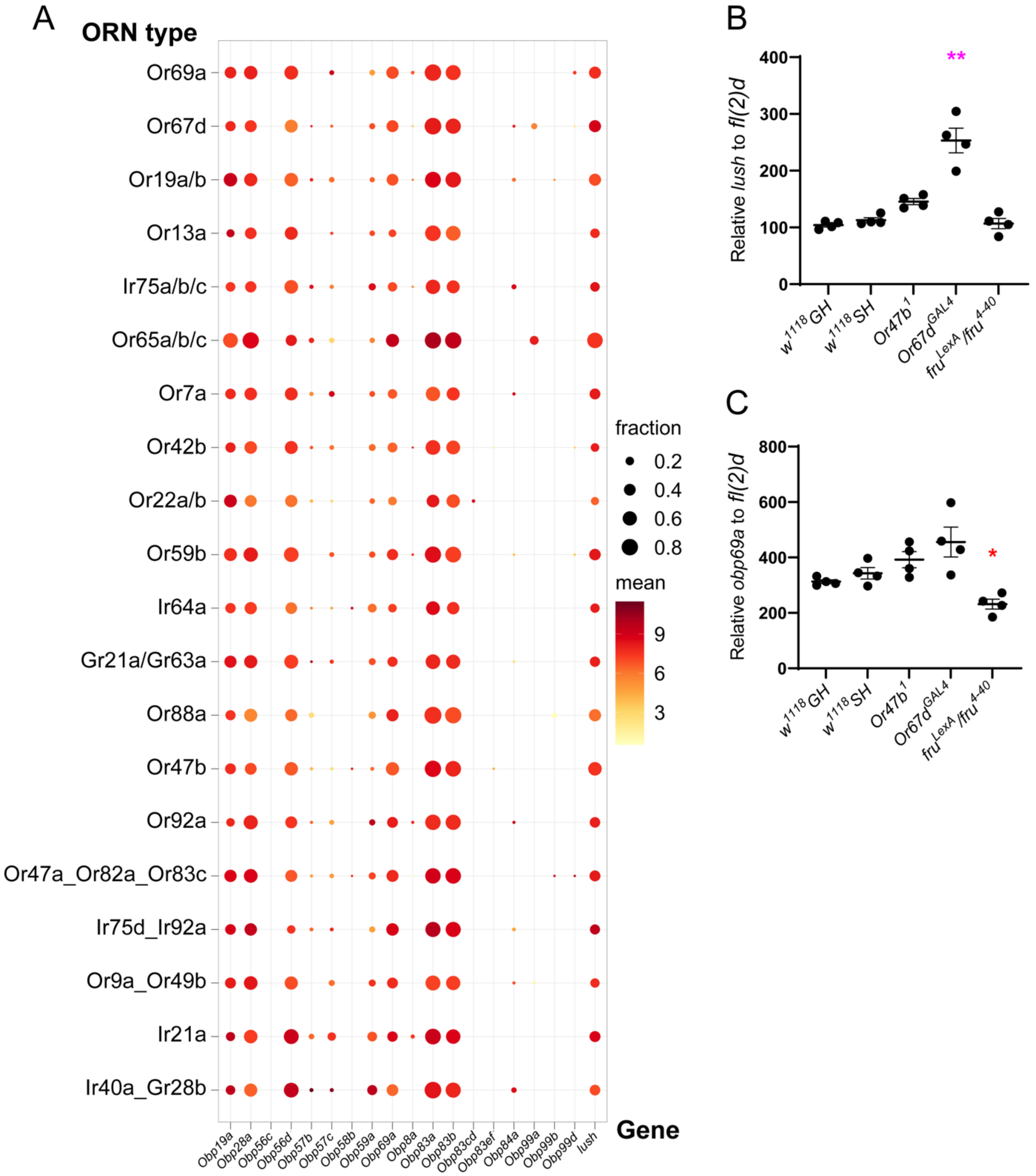

Figure S5. Validation of *Obp* expression across ORN classes and in mutants. (A) ORN class-specific expression of *Obps* based on single-cell RNA-seq datasets from the adult ORNs (MCLAUGHLIN *et al.* 2021)**.** The size of each circle indicates the fraction of positive cells ($\log_2$(CPM+1)>0.5) and color intensity indicates the mean expression

(log_2(CPM+1)) of all positive cells. **(B-C)** Quantitative RT-PCR validation of *lush* **(B)** and *Obp69a* **(C)** expression from antennae of grouped and socially isolated wild types, *Or47b* mutants, *Or67d* mutants, and fru^M mutants normalized to *fl(2)d*. One-way ANOVA was used for significance test, followed by multiple comparisons (compare other groups to w^{1118} *GH*). * $p<0.05$, ** $p<0.01$, *** $p<0.001$, **** $p<0.0001$. not significant if no * labeled.

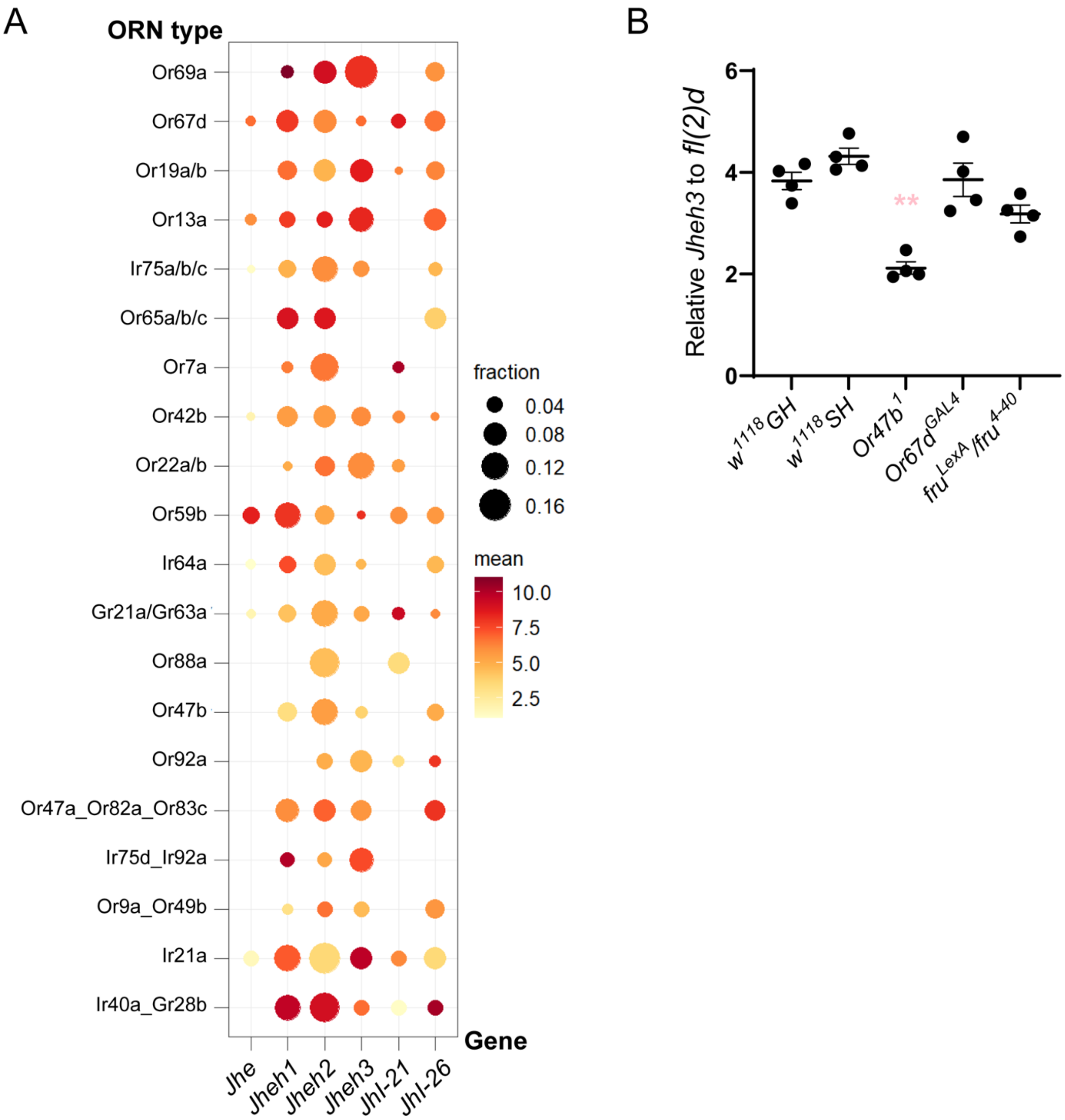

Figure S6. Validation of hormone-related gene expression across ORN classes and in mutants. (A) ORN class-specific expression of juvenile hormone regulators based on single-cell RNA-seq datasets from the adult ORNs (McLaughlin *et al.* 2021). The size of each circle indicates the fraction of positive cells ($\log_2$(CPM+1)>0.5) and color intensity indicates the mean expression ($\log_2$(CPM+1)) of all positive cells. **(B)**

Quantitative RT-PCR validation of *Jheh3* expression. Relative expression of *Jheh3* from antennae of grouped and socially isolated wild types, *Or47b* mutants, *Or67d* mutants, and fru^M mutants normalized to *fl(2)d*. One-way ANOVA was used for significance test, followed by multiple comparisons (compare other groups to w^{1118} *GH*). * $p<0.05$, ** $p<0.01$, *** $p<0.001$, **** $p<0.0001$. not significant if no * labeled.

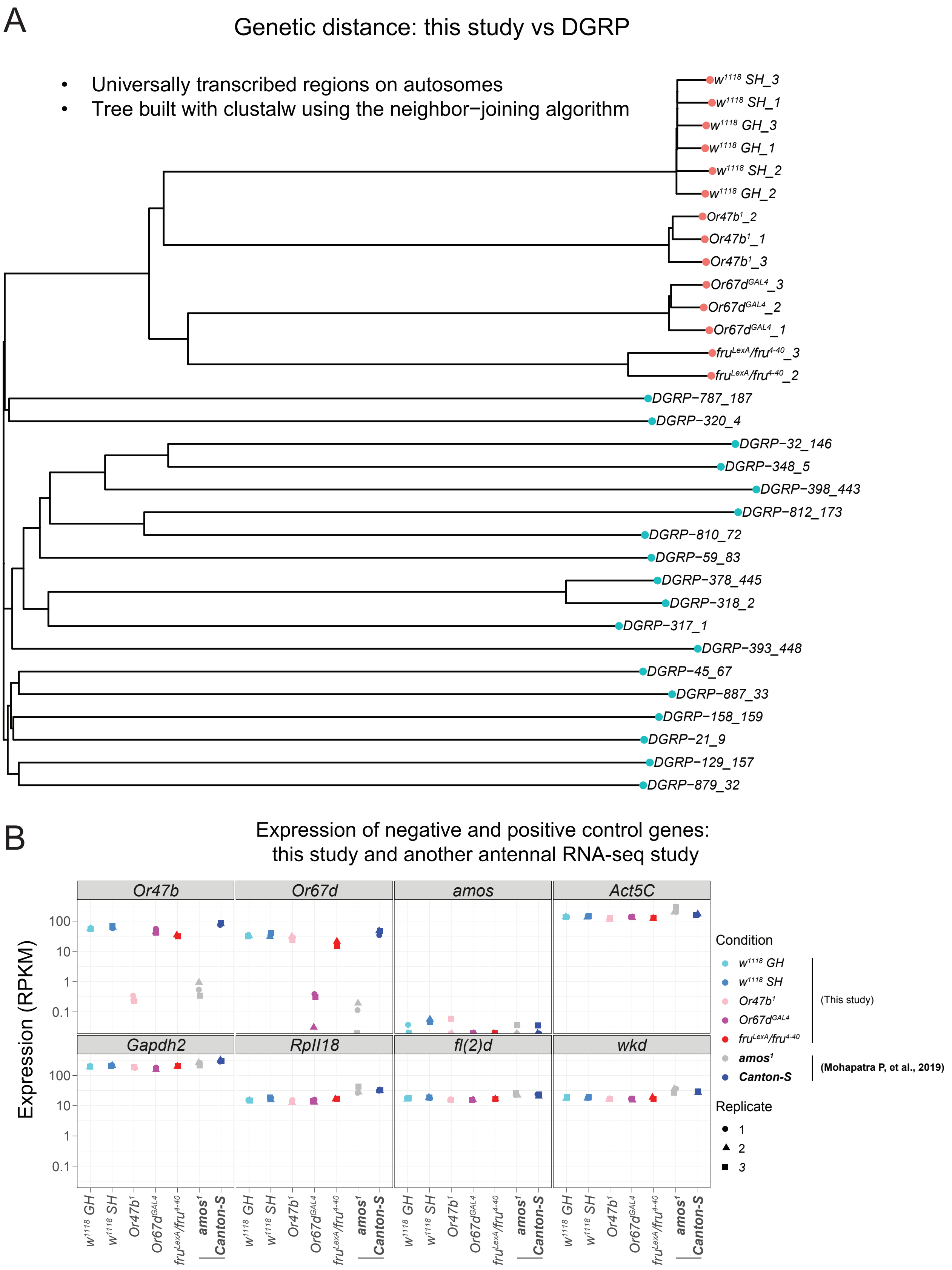

A
Genetic distance: this study vs DGRP
• Universally transcribed regions on autosomes
• Tree built with clustalw using the neighbor−joining algorithm
w^{1118} SH_3
w^{1118} SH_1
w^{1118} GH_3
w^{1118} GH_1
w^{1118} SH_2
w^{1118} GH_2
$Or47b^{1}$_2
$Or47b^{1}$_1
$Or47b^{1}$_3
$Or67d^{GAL4}$_3
$Or67d^{GAL4}$_2
$Or67d^{GAL4}$_1
fru^{LexA}/fru^{4-40}_3
fru^{LexA}/fru^{4-40}_2
DGRP−787_187
DGRP−320_4
DGRP−32_146
DGRP−348_5
DGRP−398_443
DGRP−812_173
DGRP−810_72
DGRP−59_83
DGRP−378_445
DGRP−318_2
DGRP−317_1
DGRP−393_448
DGRP−45_67
DGRP−887_33
DGRP−158_159
DGRP−21_9
DGRP−129_157
DGRP−879_32
B
Expression of negative and positive control genes:
this study and another antennal RNA-seq study
Or47b
Or67d
amos
Act5C
Gapdh2
RpII18
fl(2)d
wkd
Expression (RPKM)
100
10
1
0.1
Condition
w^{1118} GH
w^{1118} SH
$Or47b^{1}$
$Or67d^{GAL4}$
fru^{LexA}/fru^{4-40}
(This study)
$amos^{1}$
Canton-S
(Mohapatra P, et al., 2019)
Replicate
1
2
3
w^{1118} GH
w^{1118} SH
$Or47b^{1}$
$Or67d^{GAL4}$
fru^{LexA}/fru^{4-40}
$amos^{1}$
Canton-S

Figure S7. Examining the background effect of different genetic lines used for RNA-seq analysis in this study. (A) Genetic distance among our samples and 18 randomly selected Drosophila melanogaster Genetic Reference Panel (DGRP) lines. DNA sequences of lines used in this study were analyzed from universally transcribed regions on autosomes of the RNA-seq samples. Phylogeny tree was built with clustalw using the neighbor−joining algorithm (see method section for details). **(B)** Expression of the negative and positive control genes among our samples and two additional antennal samples from different labs and genetic backgrounds. *Or47b* and *Or67d* genes are not expressed in *amos* mutants; *amos* gene is not expressed in adult antenna. All other "housekeeping genes" used in this study, All other "housekeeping genes" used in this study, *Act5C*, *Gapdh2*, *RpII18*, *fl(2)d*, and *wkd*, are unchanged between *amos*^{1} mutants and *Canton-S* wildtype strains.

Methods

Genetic distance analysis of samples used in this study and Drosophila melanogaster Genetic Reference Panel (DGRP) lines

We randomly picked 18 DGRP lines, downloaded their DNA sequences from (HUANG *et al.* 2014), and mapped to the reference genome. These uniquely mapped alignments, and transcribed regions mapped from RNA-seq data of this study, were used to jointly call variants in VCF format via Freebayes (GARRISON AND MARTH 2012) using standard filters. To improve time economy, bedtools (QUINLAN AND HALL 2010) was used to restrict variant calling to those regions which have nonzero coverage in all samples. vcftools (DANECEK *et al.* 2011) was used to filter the called variants, retaining only biallelic SNPs with no missing calls. For each sample in a given VCF, bcftools (DANECEK *et al.* 2021) was used to extract and concatenate the variable alleles into a single sequence. Each site contains two alleles, one for each strand, and these were simply concatenated, i.e., each site contributes two nucleotides. These concatenations were combined in a single FASTA file and treated as a multiple sequence alignment (MSA). Genetic distance was then computed for the MSA using clustalW2 (LARKIN *et al.* 2007). The resulting tree was visualized with treeio (WANG *et al.* 2020) and ggtree (YU 2020).

Examine the control gene expression in additional antennal RNA-seq samples

We analyzed the previously published RNA-seq datasets (MOHAPATRA AND MENUZ 2019) using the same pipeline described in the main text. This dataset contains antennal RNA samples from 3-5-day-old *amos*[1] mutant and *Canton-S* wild-type flies, three biological

replicates each. The whole RPKM matrix of each replicate is in Table S5. We then plotted the expression of eight control genes used in Figure 1D of these two additional genetic backgrounds together with all five groups of this study in Figure S7B.

References

Danecek, P., A. Auton, G. Abecasis, C. A. Albers, E. Banks *et al.*, 2011 The variant call format and VCFtools. Bioinformatics 27**:** 2156-2158.

Danecek, P., J. K. Bonfield, J. Liddle, J. Marshall, V. Ohan *et al.*, 2021 Twelve years of SAMtools and BCFtools. Gigascience 10.

Garrison, E., and G. Marth, 2012 Haplotype-based variant detection from short-read sequencing. arXiv preprint arXiv:1207.3907.

Huang, W., A. Massouras, Y. Inoue, J. Peiffer, M. Ramia *et al.*, 2014 Natural variation in genome architecture among 205 Drosophila melanogaster Genetic Reference Panel lines. Genome Res 24**:** 1193-1208.

Larkin, M. A., G. Blackshields, N. P. Brown, R. Chenna, P. A. McGettigan *et al.*, 2007 Clustal W and Clustal X version 2.0. Bioinformatics 23**:** 2947-2948.

McLaughlin, C. N., M. Brbić, Q. Xie, T. Li, F. Horns *et al.*, 2021 Single-cell transcriptomes of developing and adult olfactory receptor neurons in drosophila. eLife 10.

Mohapatra, P., and K. Menuz, 2019 Molecular Profiling of the Drosophila Antenna Reveals Conserved Genes Underlying Olfaction in Insects. G3 (Bethesda) 9**:** 3753-3771.

Quinlan, A. R., and I. M. Hall, 2010 BEDTools: a flexible suite of utilities for comparing genomic features. Bioinformatics 26**:** 841-842.

Wang, L. G., T. T. Lam, S. Xu, Z. Dai, L. Zhou *et al.*, 2020 Treeio: An R Package for Phylogenetic Tree Input and Output with Richly Annotated and Associated Data. Mol Biol Evol 37**:** 599-603.

Yu, G., 2020 Using ggtree to Visualize Data on Tree-Like Structures. Curr Protoc Bioinformatics 69**:** e96.
